# Touching at a distance: the elaboration of communicative functions from the perspective of the interactants

**DOI:** 10.3389/fpsyg.2024.1497289

**Published:** 2024-12-18

**Authors:** Robin Héron, Stéphane Safin, Michael Baker, Zhuoming Zhang, Eric Lecolinet, Françoise Détienne

**Affiliations:** ^1^i3, UMR-9217 CNRS Télécom Paris, Institut Polytechnique de Paris, Palaiseau, France; ^2^LTCI, Télécom Paris, Institut Polytechnique de Paris, Palaiseau, France

**Keywords:** mediated social touch, interactive alignment, common ground, social context, remote communication

## Abstract

Touch is an inherent part of human social interactions and the diversity of its functions has been highlighted in numerous works. Given the varied roles of touch, with technology-mediated communication being a big part of our everyday lives, research has been interested in enabling and enhancing distant social interactions with mediated touch over networks. Due to the complexity of the sense of touch and technological limitations, multimodal devices have been developed and investigated. In this article, we explore the use of mediated visual touch in distant social interaction. Adopting an interactionist and collaborative approach to human communication, we focus on the communicative functions of distant touch behaviours which interactants co-elaborate throughout their mediated interactions. For this purpose, we conducted an exploratory study placing five romantically involved couples in interaction, where each discussed shared biographical events via a video call, using mediated touch devices (producing vibration and coloured lights). Their interactions were recorded, and excerpts were presented to participants in interviews using a collective confrontation technique (participants are confronted with a recording of their activity and encouraged to comment on it). This technique allows a better understanding of the participants’ points of view on their use of the touch devices in context. Through analysis of the interviews, our results highlight: (1) a variety of visual-touch functions with a redistribution of functions mostly supported by other modalities of communication in face-to-face interactions, such as illustrating aspects of the ongoing conversation; (2) the visual-touch characteristics as well as the verbal, paraverbal and non-verbal indicators of the interactional context considered by the participants to make sense of the stimuli and; (3) the multifactorial and dynamic aspects of the co-elaboration process of the visual-touch functions, reaffirming the role of interactional context, combined with cultural and biographical knowledge, in the meaning making.

## 1 Introduction

Social touch—touch behaviours occurring within social interactions, such as handshaking, hugging, kissing on the cheeks, patting the shoulder, etc.—supports a wide range of functions in human social life, and it is through the context of interaction that interactants can determine the meaning of a touch (e.g., van Erp and Toet, 2015; Jones and Yarbrough, 1985).

Given the diverse roles of touch in our social interactions, especially with regard to affective communication, interest in its integration into technology-mediated interactions is growing (van Erp and Toet, 2015), not least because of globalisation that leads to more individuals living distant in space, separated from family and friends (Janta et al., 2015; Ryan et al., 2015). Most studies focus on the emotional meaning of mediated touch in restricted experimental settings, where subjects are asked to judge the emotions expressed by discrete touching events, reporting greater feelings of connectedness, as well as the communication of several affects with various mediated touch devices (e.g., Bailenson et al., 2007; Rantala et al., 2013; Tsalamlal et al., 2013). In order to enhance mediated communication and overcome the limitations of current technology, multimodal devices have been investigated. For instance, researchers in pseudo-haptics are able to simulate social touch and elicit a feeling of presence by combining visual and auditory cues (Desnoyers-Stewart et al., 2023). Regarding the integration of additional modalities to mediated touch devices, research on visuo-tactile stimuli (combining visual and tactile cues through the means of a technological device, referred to as “visual-touch” in the rest of the article) has shown enhanced emotional communication in laboratory situations (Wilson and Brewster, 2017; Zhang et al., 2019). Exploring mediated touch in more complex interactional settings, a few studies of mediated touch have allowed for the observation of functions of touch in social interaction, such as *turn-taking*—e.g., to help change who has the floor—and co-verbal touch functions such as *emphasis*—the interactants use touch devices to emphasise certain words or utterances (Chang et al., 2002), as well as the construction of idiosyncratic meanings over time (Park et al., 2013). These studies allow for the observation of some mediated touch functions, though their methodological frameworks lack a clear theoretical background to address the understanding of mediated touch in interaction, especially the role played by the verbal interaction in the interactants’ co-elaboration of the touch functions.

Our research aims to go further, combining the use of a multimodal device and a naturalistic interactional context. We explore visual-touch and how its functions are co-elaborated in interaction. The originality of our exploratory study of visual touch is to adopt a collaborative model of human interaction, according to which meaning emerges from social interaction. This can be distinguished from the classic encoding-decoding model more widely followed in HCI (e.g., Bailenson et al., 2007; Rantala et al., 2013; Wilson and Brewster, 2017) that posits a univocal relation between the form of touch (e.g., a stroke with a certain intensity) and its function (e.g., communicating a specific emotion).

Our results highlight: (1) 12 functions of mediated touch falling into three main dimensions of interactions, with a redistribution of functions across the modalities of communication in distant mediated interactions—for instance, we observed an illustration function (interactants using the device to illustrate physical, emotional or conceptual aspects of their stories) mostly supported by co-speech gestures in face-to-face interaction; (2) several indicators drawn from visual-touch characteristics and the context of the interaction to make sense of the stimuli; (3) the co-elaboration process relying on these indicators, reaffirming the role of interactional context, combined with cultural and biographical knowledge, in meaning making.

## 2 Related work

### 2.1 The functions of social touch in face-to-face interactions

Social touch plays an important role in the communication of emotions in daily life. Jones and Yarbrough (1985) highlighted Positive Affect touches, including the communication of affection and support, which has been studied extensively. For instance, Hertenstein et al. (2006, 2009) investigated the communication of affects through touch, using pairs of participants. Their results indicate that it is possible to communicate distinct emotions through touch, including for instance anger, sadness, disgust, love, and sympathy. Besides the mere communication of affects, touch helps to maintain and negotiate social relationships. In social interactions, touch facilitates mutual understanding as interactants can emphasise certain elements of the verbal modality (words, sentences) or modulate the tone or mood of the interaction by introducing a playful dimension (e.g., Jones and Yarbrough, 1985, Knapp, 1978).

In general, social touch shows multiple beneficial effects on health and well-being. Several studies report that physical contact (e.g., holding hands, hugging) with a close relative helps reducing pain, stress and blood pressure (Ditzen et al., 2007; Grewen et al., 2003; Master et al., 2009); touching with non-relatives also has beneficial effects on heart rate, stress levels and inflammation (Henricson et al., 2008; Thomas and Kim, 2021; Whitcher and Fisher, 1979). Therefore, the lack of touch may be detrimental to human health (Floyd, 2014). However, touch behaviours have decreased in recent years, especially with the Covid-19 pandemic (Field et al., 2020), leading to challenges for establishing new touch practices (Zhang et al., 2021). In that regard, mediated social touch can be seen as a way to overcome the lack of actual social touch, raising the questions of how to design social touch devices and for what purpose, as discussed by Jewitt et al. (2021).

### 2.2 Mediated social (visual-)touch communication

Aiming to enhance computer-mediated communication with regards to affective aspects, research has investigated ways to convey touch at a distance. It appears that mediated social touch can also have a positive effect on health and well-being. When confronted with a sad emotion, mediated social touch in the form of warmth and vibration can help to mitigate participants’ sadness responses by reducing heart rate (Cabibihan et al., 2012). Furthermore, it has been shown that mediated social touch can also reduce the level of the stress hormone cortisol (Sumioka et al., 2013). As in the case of actual social touch, mediated social touch research also largely focuses on the communication of affect—showing a variety of discrete emotions or variations in valence (i.e., emotional pleasure) and arousal (i.e., physiological arousal associated with emotion) scales (Bailenson et al., 2007; Rantala et al., 2013; Tsalamlal et al., 2013; Wilson and Brewster, 2017)—and increased feelings of connectedness (Giannopoulos et al., 2008; Nakanishi et al., 2014; Sallnäs, 2010) allowed by touch in remote situations.

The expressiveness of social touch devices is limited due to cost and technology, and mediated touch stimuli can be difficult to discriminate from one another (Zhang et al., 2019). In that regard, researchers have explored the use of multimodal signals for enhanced affective communication. In virtual reality research, for example, pseudo-haptic (a technique to simulate tactile sensation in virtual environments through other modalities) notably uses the tactile-visual interaction by combining the sensorimotor actions of the user with visual feedback (Lécuyer, 2009). Recent work suggests that it is possible to use a combination of visual and auditory cues to simulate social touch between interactants in a virtual environment and elicit a feeling of presence (Desnoyers-Stewart et al., 2023). Vision appears to have a strong cross-modal interaction with touch (Gallace and Spence, 2010) and several studies highlight increased haptic spatial perception and tactile acuity with co-occurrent visual cues (Eads et al., 2015; Newport et al., 2002). Both the perception speed and accuracy of touch are improved with additional visual cues, and spatially congruent visual cues can affect tactile perception (Mancini et al., 2014).

Researchers such as Wilson and Brewster (2017), as well as Zhang et al. (2019), studied the integration of colours into touch devices, since this has been shown to influence affective communication (Valdez and Mehrabian, 1994; Suk and Irtel, 2008; Wilms and Oberfeld, 2018) and touch perception (Simner and Ludwig, 2012). Their results suggest broadened possibilities in affective communication when using multimodal devices combining tactile cues through vibrations, visual cues using colours (Wilson and Brewster, 2017) and congruent visual patterns (Zhang et al., 2019).

### 2.3 Context matters, social interaction even more

Most mediated touch studies have been conducted under strict experimental conditions, without actual interaction between the person touching and the person being touched, with the objective of correlating specific forms to specific meanings. For instance, Wilson and Brewster (2017) presented their stimuli and asked the participants to adjust cursors on a computer screen for two scales (emotional arousal and valence), without human-human interaction. However, researchers have long pointed out the limits of considering only the tactile features of social touch (Jones and Yarbrough, 1985; van Erp and Toet, 2015).

Aiming for a finer understanding of the construction of the meaning of affects conveyed through mediated touch, some researchers integrated contextual cues in their experimental protocols to investigate how they could alter the meanings of touch behaviours. Results suggest that textual and facial cues can modulate the perceived emotion of mediated touch behaviours (Ipakchian Askari et al., 2020; Teyssier et al., 2020). Price et al. (2022) highlighted the crucial role of context for the elaboration of meaning. In two experimental studies, they showed that mediated social touch using pressure and temperature can convey a myriad of emotions between people who have close relationships (e.g., partners, friends), depending on the sensorial characteristics of touch, and on its context of occurrence (e.g., the relationship of the interactants, textual context), which helps to “negotiate the ambiguity.” The importance of the context surrounding both interactants is also reaffirmed by the Remote Social Touch framework proposed by Alsamarei and Şener (2023). Investigating touch in everyday life between couples, Sailer et al. (2024) underline “the complexity of interpersonal touch in everyday life.” Their results indicate that the interaction partner, situational characteristics and needs fulfilment, such as relatedness, are better determinants of the valence of a touch experience, in comparison with its physical characteristics.

Most of the studies of mediated touch in interaction consist of a controlled environment where researchers identify the effect of one or more factors on a small number of dependent variables (e.g., feelings of presence, helping behaviour, task success; e.g., Haans, and IJsselsteijn, W. A., 2009; Nakanishi et al., 2014). However, a small number of studies explore mediated touch in social interactions under certain tasks or conditions (e.g., making a list of objects dedicated to survival, communicating exclusively by audio and touch, minimising the use of speech), thus paving the way to identifying potential functions of mediated social touch in interaction.

Chang et al. (2002), used the ComTouch device (connected smartphones allowing the transmission and reception of vibrations) and audio communication, following an experimental protocol. Their study highlighted four categories of mediated touch functions: *emphasis* (highlighting certain points in the message); *turn taking* (to make the exchange more fluid); *mimicry* (a game of imitation with vibration patterns); and *coding*. The latter is particularly used when speech is limited, where creating a code allows speakers to exchange “yes/no” responses or to count. The work of Park et al. (2013) with the POKE system (a phone with an area that swells in response to pressure on the remotely connected phone) through a longitudinal study with three couples in a long-distance relationship, showed the emergence of a shared code (e.g., “I love you” represented by two very weak touches; “it’s annoying” represented by six strong and fast touches) amongst couples.

These studies give an insight into functions of mediated touch and their elaboration throughout social interaction. Our aim is to go further, hence our need for a clear theoretical and methodological framework to comprehend how social visual touch functions are co-elaborated in interaction.

### 2.4 An interactive-collaborative approach to the study of mediated touch in interaction

As suggested by the manifesto of Jewitt et al. (2021), in order to design touch we believe that it is necessary to understand mediated social touch throughout the course of an interaction. Therefore, in line with Huisman’s (2022) perspective, our position is that the analysis of social interactions is better suited to explaining social touch interactions and “how we understand each other in day to-day interactions” (*ibid.*, p.3). In a verbal interaction, speaker and addressee co-construct the interaction itself; even while listening, interactants actively regulate the interaction through numerous behaviours signalling their degree of attention and (lack of) understanding (Kerbrat-Orecchioni, 1996). We see inter-action as a series of interdependent actions, verbal or not, which mutually influence each other, involving two or more interactants (Baker, 2004; Olry-Louis, 2011) or as Goffman stated, interaction “may be roughly defined as the reciprocal influence of individuals upon one another’s actions when in one another’s immediate physical presence [in our case, telepresence]” (Goffman, 1959, p.15).

According to a collaborative theory of human communication, the function of touch is contextual in an extended sense, taking into account the dynamic evolution of the interaction context. Whereas this paradigm has been extensively used to understand the communicative functions of various modalities of communication (e.g., verbal, gestural) it has not been mobilised to understand the functions of the tactile modality.

The co-elaboration of the functions of touch can be understood in terms of the processes of: (1) interactive alignment (Garrod and Pickering, 2009)—automatic alignment of para-verbal behaviour in the interaction (e.g., alignment of posture or speech rate)—and (2) grounding (Clark and Brennan, 1991; Clark and Schaefer, 1989)—the interactive process by which interactants exchange evidence about what they do (not) understand over the course of a conversation, as they accrue common ground by a collaborative effort. In these theoretical frameworks, the co-elaboration of the associations between forms and meanings is observed at the micro level with *ad hoc* constructions, and not only with associations that are stable in time, observed at the macro level. For instance, when two persons are having a conversation about a child and the first interactant utters “He’s still healthy” followed by the second interactant who utters “He’s still walking around,” both forms (healthy and still walking) are locally associated with the same meaning (Brône and Zima, 2014).

Vion (1992) proposes to categorise the different functions of verbal interactions through three main dimensions: *interaction management*, *meaning making*, and *relationship building*. In the following paragraphs, we briefly review verbal, para-verbal and non-verbal functions according to these dimensions.

*Interaction management*—through the verbal modality, interactants can give feedback, structure the interaction or manage turns with dialogue control acts (Bunt, 1994) or turn-taking acts (Traum and Hinkelman, 1992). Non-verbal and paraverbal behaviours also helps, as in the initiation and closing of the interaction (Floyd et al., 2000; Kendon, 1990; Knapp et al., 1973), turn management (Duncan, 1972; Knapp, 1978) or speech segmentation (Burgoon et al., 2016; Quek et al., 2002) with gaze, hand gesture, or prosody.

*Meaning making—*by their verbal behaviour such as informative acts (e.g., question, inform, correction; Bunt, 1994), grounding (e.g., repairs, continuers, acknowledgments) and core speech acts (e.g., inform, suggest; Traum and Hinkelman, 1992), interactants co-elaborate the meanings of their interventions. Through prosody, the interactants can make more precise or modulate meanings, notably in the case of humour or irony (Attardo et al., 2003; Bryant and Fox Tree, 2005), and add emphasis to the verbal content (Chieffi and Ricci, 2005; Quek et al., 2002). Iconic gestures—forms related to meanings—can also accompany speech (Hadar and Butterworth, 1997; McNeill, 1992).

*Relationship building*—paraverbal and nonverbal behaviours such as emotional communication (Buck et al., 1992) with facial expressions (Ekman et al., 1972), prosodic and paraverbal cues (Banse and Scherer, 1996) or touch (Hertenstein et al., 2006) play an important role in the co-construction of relationships through the course of their interactions.

As for verbal interactions, we posit that mediated (visual-)touch functions are co-elaborated in action by the interactants taking context into account (un)consciously. They can be analysed through the three aforementioned dimensions.

Our study aims at understanding *how the functions of visual-touch emerge in interaction*. For that purpose, we address three research questions:

- RQ1: How does the specificity of visual-touches influence the elaboration of their functions?
- RQ2: What are the indicators used by interactants to elaborate the functions of their visual-touches?
- RQ3: How do interactants reach mutual understanding of visual-touches in interaction?

**Figure 1:**
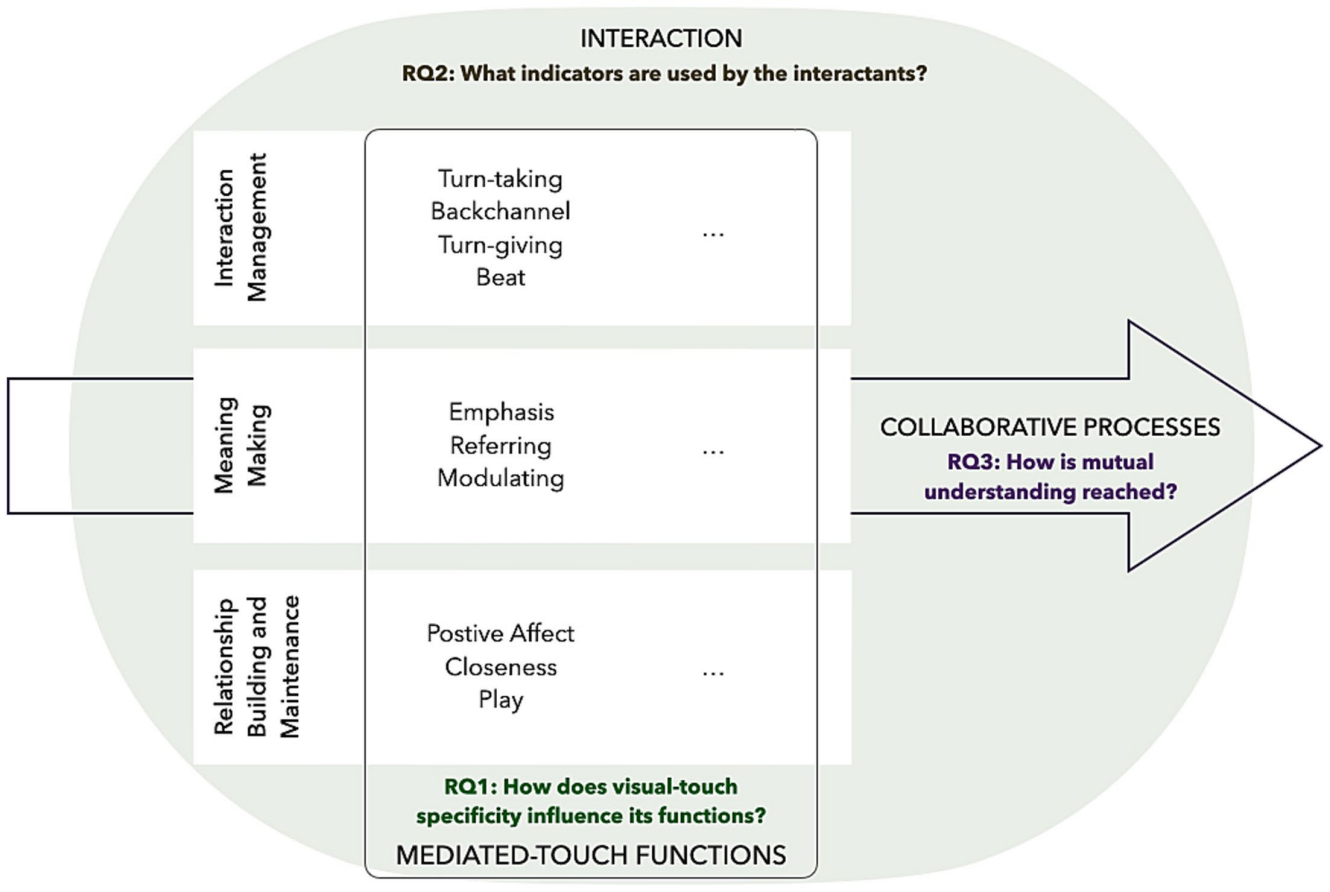
Frameworks and research question integration. The figure illustrates the framework we use to address each research question. The large round shape represents a social interaction, within which are presented the dimensions of functions (Vion, 1992) with the associated functions of mediated-touch (highlighted by Héron et al., 2022) as well as empty spaces for possible specific functions of visual-touch (or alternative mediated-touch devices). An arrow runs through the interaction to depict the dynamic and collaborative processes taking place within the interaction. The research questions (RQ) are associated with the different frameworks.

In brief, the frameworks described above scaffold our work as follows (cf. Figure 1). Tthe dimensions of interaction functions (Vion, 1992) and previously highlighted functions (Héron et al., 2022; presented in section 3.5) will enable us to understand how the characteristics of VisualTouch influence its use (RQ1). The understanding of the interaction as a series of interdependent multimodal actions (Kerbrat-Orecchioni, 1996; Baker, 2004) will guide our identification of the indicators used to elaborate the functions of visual-touch (RQ2). Finally, theories of human communication (Garrod and Pickering, 2009; Clark and Brennan, 1991), positing that meaning is co-elaborated through interactive and collaborative processes, will frame our approach to the mutual understanding of visual-touch functions (RQ3).

## 3 Method

### 3.1 A mediated-touch device: VisualTouch

The device we used in our research is based on multimodality (tactile and colour stimuli) which has been previously acknowledged as being able to communicate emotion combined in mediated touch devices (see section 2.2.; Huisman and Darriba Frederiks, 2013; Wilson and Brewster, 2017). A first prototype of this device (combining vibration and coloured visual patterns) has previously demonstrated its possibilities for affective communication in a laboratory experiment (Zhang et al., 2019).

The current prototypes used in this study were slightly revised in order to work wirelessly in interactional contexts. They comprise two superimposed layers: 60 multicoloured LEDs and 10 servomotors each moving a small rod (1 servomotor for 6 LEDs, instead of the 60 vibration actuators in the original design which were leading to lags over time). The idea behind this two-layered device is for the visual cues to alleviate the ambiguity of the tactile stimuli alone. Thus, every stimulus received combines visual and tactile sensations congruently mapped on the forearm (participants wore the device on the arm of their choice). The competition between neural representations and the recruitment of attentional resources results in a visual dominance effect (Hartcher-O’Brien et al., 2008), so that users generally do not perceive small conflicts between visual and tactile cues (Ramachandran and Rogers-Ramachandran, 1996). The two layers and all the circuitry are integrated into a 3D-printed case, and the LEDs are covered by a translucent screen. Each device has a Wi-Fi antenna and a battery, enabling it to operate completely independently. The devices are automatically connected to a Wi-Fi router, to which the smartphones used to control them are also connected.

**Figure 2:**
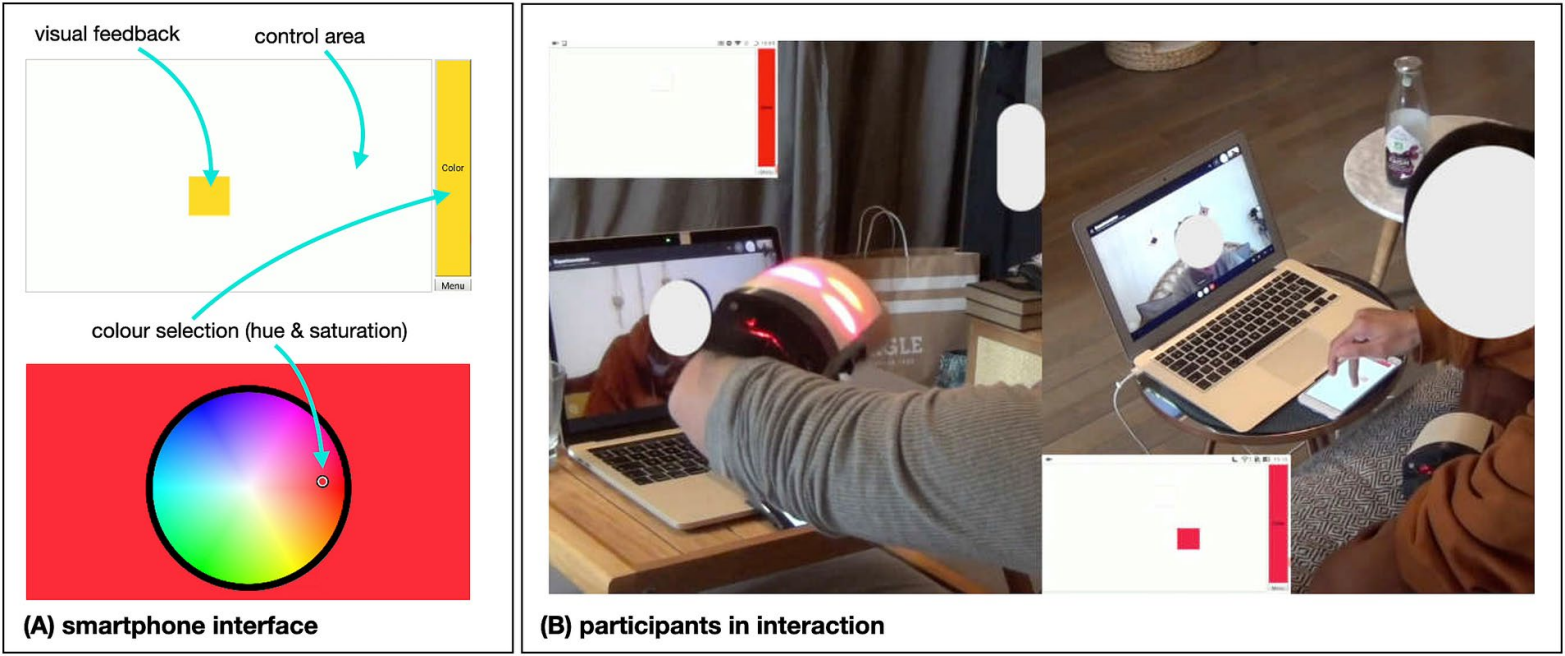
VisualTouch device. **(A)** Shows the interface presented on the smartphone through which the participants can create/send visual-touch stimuli. **(B)** Shows an excerpt of the interaction of a couple. On the left, the visual effect on the forearm of the interactant is produced by the use of the smartphone of the interactant on the right (due to a decay effect on the visual and short transmission delay, three red dots can be seen on the screen while there is only one on the smartphone). For a better understanding, short video excerpts are available online in Supplementary Videos 1, 2.

The devices are controlled by a web application (accessible via smartphone) that allows the users to select the colour and draw visual-touches on a white interface. In order to select a colour, the participant has to first touch the coloured zone on the right of the screen, then choose the colour in a circular colour picker. The hue and saturation can be adjusted in this way. The selection is confirmed with a dot appearing where the selection was registered and the background changing colour before going back to the interaction screen. Visual feedback on the screen confirms to the user which areas have been touched and with what colour (Figure 2).

### 3.2 Participants

We recruited cohabiting couples given that people in close relationships use touch more in day-to-day life (Heslin and Boss, 1980; Smith and MacLean, 2007) and are able to co-elaborate a common understanding of mediated touch (Brown et al., 2009, Park et al., 2013; Price et al., 2022). This allowed us to observe many touch behaviours covering a wide range of functions.

Participants were recruited through word of mouth, amongst friends and friends of colleagues. The final sample was composed of five cohabiting couples. None of the participants were colourblind. They neither had previously used visual-touch or mediated touch devices, and they had no knowledge of mediated touch technologies (see Table 1).

**Table 1:** Couples of participants.

Couple	Participant A			Participant B			Interaction duration	Interviews duration
C1	32 yrs	F	Production assistant	30 yrs	F	Actress	35 min	1 h 21 min
C2	28 yrs	F	Designer	30 yrs	M	Engineer	30 min	1 h 12 min
C3	28 yrs	F	Unemployed (training in real estate)	28 yrs	M	Business Consultant	21 min	1 h 09 min
C4	37 yrs	M	School teacher	30 yrs	M	Dancer	24 min	1 h 04 min
C5	29 yrs	F	Speech therapist	30 yrs	M	e-commerce manager	22 min	43 min

Each line reports information related to the couples (C1 to C5) involved in our study, with from left to right, the couple code, the age, gender and profession of each partner, the total duration of their interaction, the duration of the interview.

With a small-sized sample, we were able to deploy a complex protocol in order to frame close-to-natural remote interactions and obtain participants’ in-depth perspectives on their use of visual-touch in interaction.

### 3.3 An interactionist protocol in participants’ homes

Drawing from our interactionist theoretical framework, we developed a methodological framework to analyse touch in coherent and close to naturalistic social interactions.

**Figure 3:**
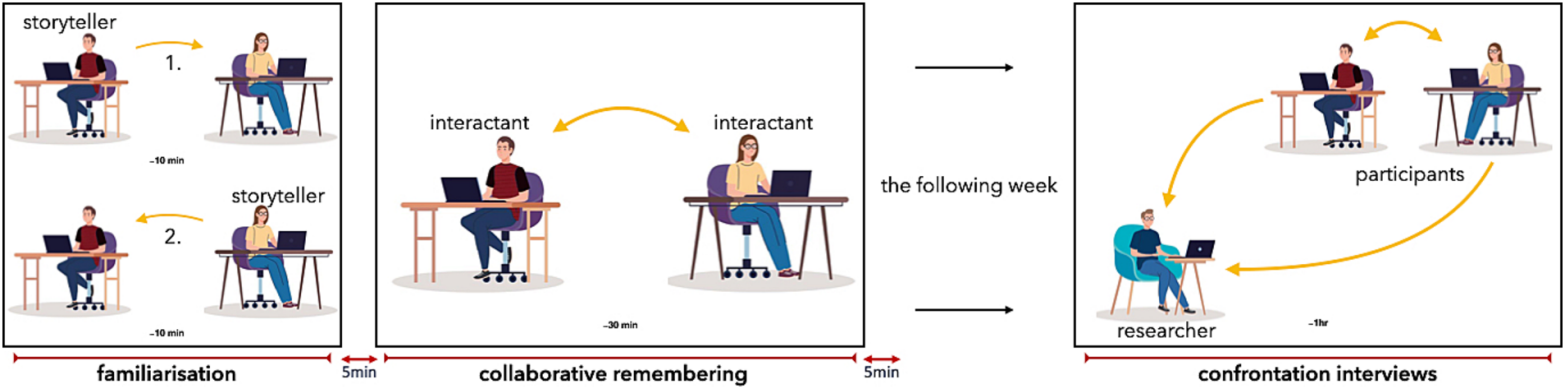
Protocol phases. Each panel represents a phase of the protocol. In the familiarisation phase, participants alternately talk about their story while the non-talking participant can respond through touch. During the collaborative remembering phase, participants can talk freely as they discuss their memories. There are five-minute breaks between the phases (after the familiarisation and after the collaborative remembering) for the participants to talk about their first experience of the device. Participants are interviewed during the following week. Both participants discuss with each other and answer to the researcher’s question, the video recording of their interaction being used as a support for remembrance (the images used in the figure are from Studiogstock and are free of royalties).

The protocol consisted of three phases: familiarisation, collaborative remembering, and, in the following week, a collective confrontation interview—a technique for analysing human activity. Participants are presented with a video-recording of their activity and encouraged to comment on it (see Figure 3). Between and after the first two phases, participants were given five-minute breaks during which they were able to discuss their use of the device. The first two phases took place at the participants’ home. The interviews were conducted remotely via video call.

The participants were both equipped with the VisualTouch device (which they were asked to wear on their forearm), a laptop and a smartphone to control the touch device. Participant A’s phone was paired to Participant B’s touch device and *vice-versa*.

Before the familiarisation phase, the participants were invited to explore the device on themselves (controlling the device they wore) for around 5 min, so they could grasp how their use of the smartphone app affected the perceived stimuli. Then, during their interaction, participants used the device in a variety of way by “drawing” different patterns on the smartphone’s screen, playing with rhythm, changing colour, and supporting a variety of functions (cf. video excerpts online Supplementary Videos 1, 2; presented in more detail in section 4).

#### 3.3.1 Familiarisation phase

The participants were asked to take turns telling their life stories, focusing on highlights and positive/negative events. Each participant had a maximum of 10 min to do so. The listener’s microphone was switched off. Listeners were encouraged to react to the speaker’s story using the available modalities (webcam and touch device). The purpose of this phase was for the participants to discover the device, to feel more comfortable with being filmed and to explore the use of mediated touch in context.

#### 3.3.2 Collaborative remembering phase

Collaborative remembering activities occur in everyday life, when people relive shared memories (Maswood et al., 2019), hence our choice of this task. Furthermore, the task encourages the embodiment of memories—with the involvement of co-verbal gestures, glances, etc.—and the expression and regulation of memories. In this phase, the participants are lead to express and regulate their emotions (Alea and Bluck, 2003; Bietti et al., 2016; Bietti and Galiana Castelló, 2013; Kendon, 1986), which we believed would also encourage the use of social touch.

Prior to the day of the observation, participants were informed that they would talk about events they had experienced together based on related artefacts (e.g., a concert ticket, a museum ticket, a photograph, a book, a coffee cup). On the day of the observation, participants alternately presented their artefacts and related memories, and then continued to talk around and about these shared events. Even though the protocol is structured, the interaction of the participants in this phase was mostly free. Once the proceedings of this phase were clearly understood, participants discussed the different artefacts until they had nothing to add or wished to skip to the next one autonomously (without the researcher asking them to). Furthermore, they often discussed other subjects not related to the artefact, and were not forbidden to do so. The primary objective of this task is to be able to observe conversations that are natural and rooted in the participants’ lives, hence at no time does the researcher intervene (even if the participants discuss events not directly related to the artefacts) except when directly questioned. During this phase, participants could communicate through all modalities (i.e., oral, visual, tactile).

#### 3.3.3 Confrontation interviews: the functions of touch and their co-elaboration

In the following week, participants were interviewed on the basis of the video recordings of their interactions. The time gap between the interaction and the interview was the result of the time needed to prepare the video and of the availability of the participants. The principle of self-confrontation interviews is to question the participants with respect to traces of their actions (video and audio conversation excerpts) in order to access their points of view on what they are doing and assess underlying cognitive processes (Mollo and Falzon, 2004; Theureau, 2010). This type of interview has been largely used in the fields of psychology and cognitive ergonomics and in various settings (Cahour et al., 2016). In dynamic and multimodal settings, such as social interactions in our case, the video recording helps mediate remembering as a memory primer (Cahour et al., 2018).

In order to better grasp the way visual-touch functions are co-elaborated by the participants, we conducted collective confrontation interviews allowing for exchanges within the couples about what was understood and how. Fostering discussion between participants on their interaction, we were able to focus the interviews on the indicators enabling the participants to elaborate these functions in interaction.

Each couple was first asked to recall moments they found particularly salient with regard to their use of the VisualTouch device (moments where they thought they mutually understood their use of the device particularly well or on the contrary when they thought it did not work), then we displayed the excerpts mentioned by the participants. For each excerpt, we invited the participants to take time to relive their interaction and describe what was happening. We then used prompts to obtain more details about each of the touches, and the communicative functions they associated with them at the time, and why. Participants were free to rewatch any moments at any time during the interview.

### 3.4 Data recording

The participants’ computers were connected via a videocall software. The researcher (first author) was also connected to the call, in order to record the audio channel. During the interaction, the participants were filmed by two cameras (one for each participant). The cameras captured both the computer screen, the touch device, and the smartphone. The smartphones screens were also recorded. The views of the two cameras, the audio of the video call and the screens of the two smartphones were then merged into a single audio-video file, for each couple, to be analysed and presented in interviews.

### 3.5 Pre-testing the protocol

We conducted a pilot-study with three couples to test our methodological framework in which we demonstrated that it enables the observation of a variety of mediated touch behaviours (Héron et al., 2022). We highlighted 12 functions supported by mediated touch.

Drawing on the dimensions of social interactions proposed by Vion (1992): *interaction management*, *meaning making*, and *relationship building* (see section 2.4), our categorisation and functions descriptions are presented in Table 2. The function previously called “doodle” is considered outside of our categorisation in the research described here, as a particular form of touch related to self-touch that we call adaptors (Lefebvre, 2008).

**Table 2:** Mediated social touch functions in distant communication.

Dimensions of functions (Vion, 1992)	Functions of mediated touch (Héron et al., 2022)	Descriptions of the functions
Interaction management	Turn-taking	The addressee touches to take the turn.
	Backchannel (Continuer)	The addressee gives indications to the speaker of their attention
	Turn-giving	The speaker indicates that their turn is over with a touch.
	Beat	The speaker produces rhythmic tactile behaviours related to the prosodic structure of the speech, which do not convey any semantic information.
Meaning Making	Emphasis	Speakers tend to emphasise certain words or phrases (mostly strong emotional content) with co-occurring touch behaviours.
	Referring (Understanding)	Speakers touch when determining the object of their interaction sequence. These touches co-occur with deictic word (e.g., “that,” “the one on the right”) and gestures (e.g., pointing, turning the head), or with implicit content (e.g., participants omitting the end of a story, alluding) and its mutual understanding (e.g., “okay!,” “ah!”).
	Modulating (Playful interaction and Treading Carefully)	The speaker touches to modulate their speech so that their partner understands it to be playful, ironic or apologetic for instance.
Relationship maintenance	Positive Affect	Interactants communicate positive affects such as love, tenderness, support.
	Closeness	Interactants use the device to maintain a sense of closeness between them. Touching is often explained as mimed caressing.
	Play (Mimicry)	The interactants play with the device. This can be a question-and-answer game with the device, or to repeat the partner’s touch.
Adaptors (Doodle)		The interactant uses the device as they would manipulate a pen or a rubber band or scratch themselves for example, mainly while listening to their partners, but also while speaking.

The table presents the categories of interactions functions proposed by Vion (1992) with which the functions of mediated touch observed by Héron et al. (2022) have been matched. The last column described each function. Adaptors is a term proposed by Lefebvre (2008), corresponds to Doodle-like touches and seats outside the presented dimensions.

The results bring to light the collaborative processes of elaborating the meaning of touch, since we observed specific ways in which the device was used, depending on the couples concerned, and no clear relations between visual-touch forms and associated functions.

Our present study aims at highlighting these underlying processes of co-elaboration in interaction, as well as the specificity of visual-touch functions. Starting from the functions observed in our pilot-study, we focus on the context indicators produced and noticed by the participants during their interaction to make sense of mediated touch, as well as their degree of mutual understanding.

### 3.6 Analysis of the confrontation interviews

The interviews were transcribed in their entirety and were first analysed into three levels of discussion. Reading through the transcripts several times, the first author highlighted sections where participants were discussing their use of the device with different degrees of precision: (1) general description of their use of the device or understanding for the entirety of the interaction; (2) broad description of use and understanding within an excerpt; (3) singular visual-touch description, its functions, and understanding.

We then screened the videos of the interactions to find excerpts for each visual-touch described by the participants (level 2 and 3). For that purpose, the video recordings were annotated with Elan software (ELAN, 2022). The recordings were segmented with the protocol phases, then each identified excerpt was annotated (with the timing, duration and colour of the stimuli) and given a name to facilitate future watching and support our understanding of the participants’ descriptions. This annotation also helped identify relevant examples for illustration purposes.

In total we were able to clearly identify 71 instances of visual-touch behaviours within the video-recorded interactions, based on the participants descriptions in interviews: 37 specific behaviours (level 3) and 34 broad descriptions (level 2), for a total of 71 visual-touch behaviours and associated functions and indicators (see Table 3). Information about their general use of the device (level 1) helped us understand the way each couple used the device overall and verbatims are presented in the results in order to illustrate them.

**Table 3:** Visual-touch events reported in the interviews.

Excerpts	C1	C2	C3	C4	C5	Total
Familiarisation	0	0	0	3	0	3
Memory n°1 A	0	0	0	0	0	0
Memory n°1 B	2	2	3	3	0	9
Memory n°2 A	3	1	4	3	2	13
Memory n°2 B	7	2	4	0	2	16
Memory n°3 A	3	7	2	2	1	14
Memory n°3 B	2	6	2	2	3	16
Total	17	18	15	13	8	71

The table presents the number of visual-touch event reported during the interviews for each couple (C1 to C5) and matched with the phases of the interaction.

The analysis of the interviews was conducted mainly on the basis of the highlighted interview transcripts. The coding scheme was elaborated in an interactive way by the four authors of the paper. This iterative process in refining the categories over time aimed for a better reliability of the results. When stabilized, the first author coded the whole corpus on the basis of the elaborated codebook.

The first step was to annotate the 71 visual-touch behaviours highlighted. While reading the verbatims multiple times, we were attentive to the expressed functions, the description of the visual-touch characteristics, the touch initiator, the elements supporting the understanding of the functions as expressed by the participants such as reference to the verbal, paraverbal and nonverbal elements, or any other descriptions of the context given by the participants.

In order determine the functions of the visual-touch (RQ1), we merged the categories proposed by Héron et al. (2022) and Vion (1992) (as presented in section 3.5). We were thereby able to categorise visual-touch functions on the basis of the participants descriptions in interview.

The analysis of the indicators used by the participants to co-elaborate the functions (RQ2), followed an inductive approach starting from the four types of indicators: visual-touch cues (characteristics of the sent stimuli), verbal (communication through spoken words), paraverbal (paralinguistic elements of speech such as pitch, volume, lengthening) and nonverbal (elements of communication such as gestures or facial expressions) context.

Both these analyses were iterative and involved four of the authors of the paper. To keep track of the annotations and categorisation overtime, we used an Excel file.

The final codebooks of the functions and the indicators can be found in the Supplementary material (cf. Supplementary Tables 1, 2).

Concurrently, for each visual-touch behaviour, we determined, when possible, the degree to which the elaboration of the function was indeed collaborative in order to answer RQ3. For that purpose, drawing from the interviews, as well as the interaction extracts, we noted whether: (1) participants discussed the mutual understanding of the functions, (2) the reported behaviour was perceived by both interactants, as well as (3) the degree of mutual understanding.

## 4 Results

We first present the functions of the visual-touch behaviours reported by the participants, then the different categories of indicators used by the interactants with contextualised examples. Finally, we look at the relation between the shared understanding by the partners and, the reported functions or indicators.

In the following subsections, we illustrate with verbatims the functions highlighted and excerpts of the interactions. In the transcripts of the interactions, ‘.’ indicates a pause, ‘-’ a lengthening, ‘[]’ an overlap, and ‘()’ gives further paraverbal information. Touch behaviours are represented by
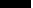
to present their occurrence and duration, information regarding their form is added underneath in *italics* when necessary.

### 4.1 Functions of visual-touch (RQ1)

The functions highlighted in Héron et al. (2022) gave us a framework of analysis for categorising those mentioned by the participants in the present study. Two additional functions are identified. First, we notice the use of the device to communicate negative affect. Second, and more interestingly, we highlighted illustrative visual-touches.

**Figure 4:**
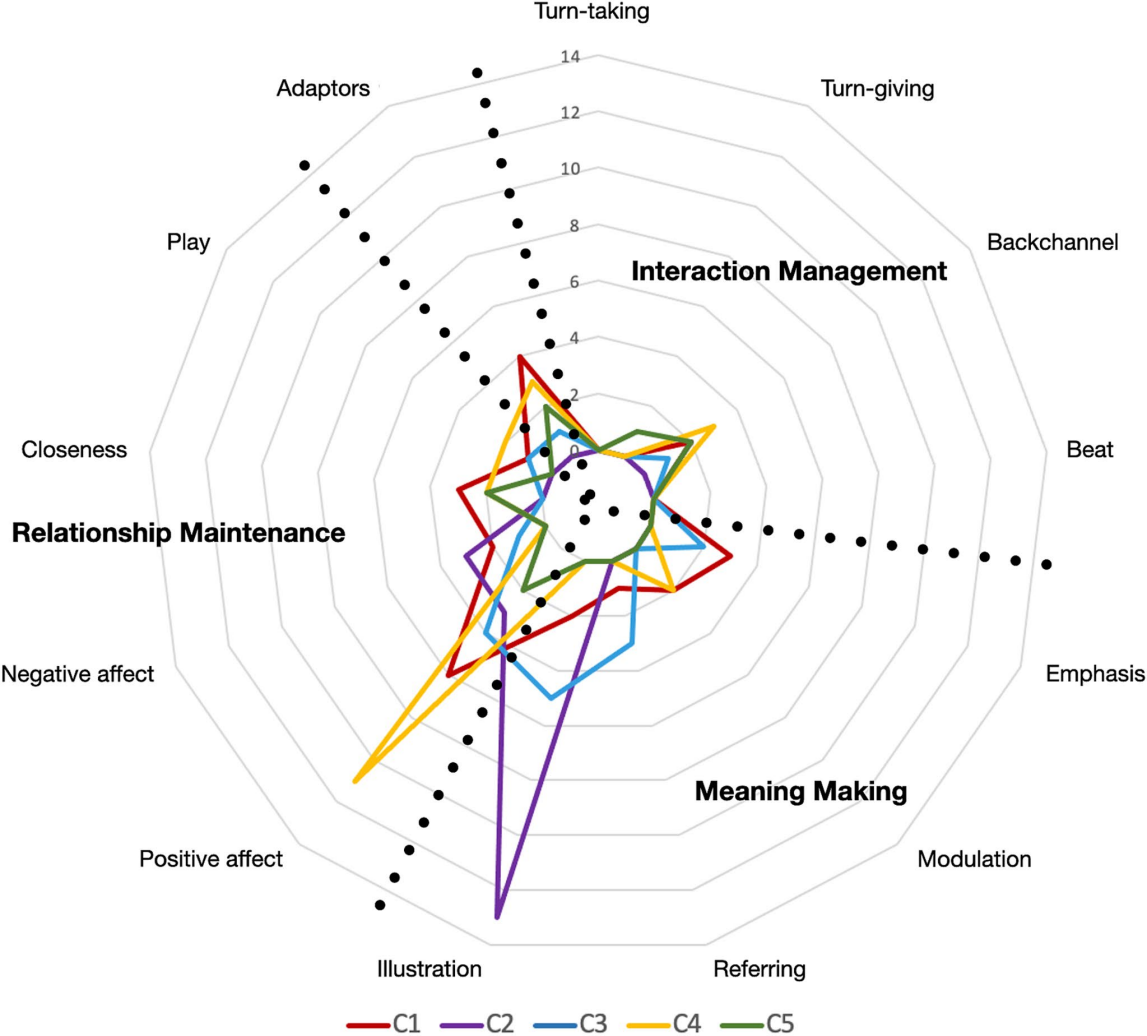
Visual-touch functions. This graph presents the distribution of the functions across couples (C1 to C5). Each coloured line represents a couple, and each concentric line represents the number of reported functions from 0 (the most central line) to 14 (the outer line). The three dimensions of functions are superimposed onto the graph.

The reported functions are highly dependent on the couple, hinting at specific co-elaboration processes. Two couples (C1 and C4) reported mainly positive affect communication functions. Two other pairs (C2 and C3) reported mainly illustration functions. For couple C5, one of the participants used the device very little (7 times over the whole interaction) for the functions of closeness and backchannel, while his partner used the VisualTouch device mainly for its adaptor function and the communication of positive affect (cf. Figure 4).

The *interaction management* category of functions is the least mentioned in the interviews. This is not surprising, since these behaviours are mostly performed unconsciously, similarly to what we observed in Héron et al. (2022). The participants do, however, report two of the functions highlighted previously: Backchannel and Turn-giving.

In terms of *meaning making*, the participants reported functions of Emphasis, Modulation, Referring, and the newly highlighted function of Illustration—the visual-touches illustrate concepts, scenes or emotions related to the stories.

As regards the functions of *relationship maintenance*, the same functions as in the pilot-study were identified. However, in this study, participants reported the communication of negative affect directed towards the partner or to the events discussed.

*Adaptors* are clearly identified by the participants, who associate them with doodling while listening on the phone for example, as well as a way to mitigate stress relative to the experimental setting or their relived memories. These self-oriented behaviours were numerous, and the context helped the interactants to understand the orientation of the visual-touch, as we will see in the following section.

### 4.2 From indicators to the co-elaboration of visual-touch functions (RQ1 and RQ2)

In this section, we present the indicators as highlighted in the interviews. Following our research questions, we are interested in two types of elements involved in the elaboration of the functions in interaction: (1) the characteristics of visual-touch behaviours (tactile and visual) and (2) the interactional context indicators (verbal, para-verbal, and non-verbal content).

The following two subsections present these elements (summarised in Table 4).

**Table 4:** Prevalence of the indicators reported by the participants.

INDICATORS		C1	C2	C3	C4	C5	Total
TACTILE	pattern		1	3	1		4
	cadence	2	8		6	1	17
VISUAL	colour	3	4	2	3		12
	illustrative aspects	3	4	2			9
VERBAL	explication	1	4	2	1	1	9
	theme	9	16	7	10		42
PARAVERBAL	alignment and prosody		1				1
	interaction role	1		1	3	5	10
	interaction time			1		1	2
NONVERBAL	gaze	2			4	1	7
	facial expression	1			1		2
	mimicry	1		2	2	1	6

The table presents the number of indicators reported by each couple (C1 to C5) and categorised in consequence, the characteristics of the stimuli (tactile and visual cues), the interaction context (verbal, paraverbal, and nonverbal elements).

#### 4.2.1 Tactile indicators

##### Patterns

The couples explain their use of the device using different patterns. They noted the distinction between dots and lines. Some also specified the direction of these lines during the interviews (e.g., upwards and downwards).


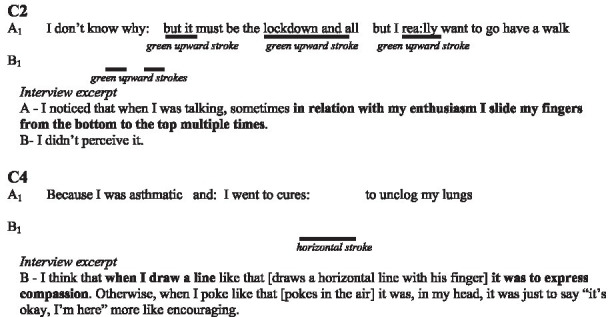


In these two examples, the indicators reported by each participant regarding the form or direction of the visual-touches were not discussed within the couples in interviews. Only one of the interactants explained how they conceived the tactile aspect of their visual-touches. We assume that this is because patterns are not perceived easily. We did not notice any evidence of mutual understanding or misunderstanding.

##### Cadence

As three couples (C1, C2, and C4) mentioned, what was mostly perceived was the rhythm, the cadence of the touches. For the participants, it was a question of discriminating between the haste and frequency of the touches. They distinguish slow, continuous touches and repeated taps of varying speed for instance, rather than their direction or form.


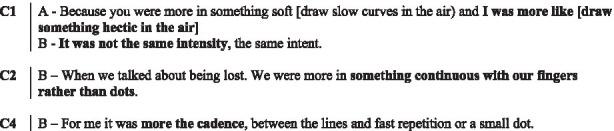


#### 4.2.2 Visual indicators

##### The colours

Couples elaborated thoroughly about their choices of colour in the interaction (for 30 behaviours out of 71).

In couple C1 and C2, each partner carefully considered the colour choice when using the device for most cases. In couple C3 and C4, only one of them considered the colour with a colour code in mind. In addition to the illustrative nature of colours in describing physical aspects of the story (e.g., yellow for the sun, green for the trees) which we will present further down, participants report familiar emotional associations. For instance, red represents love and passion, but also excitement, tension, negation, and danger; blue represents calm, well-being, appeasement and even sadness; green is associated with hope; yellow is associated with gentleness and happiness.


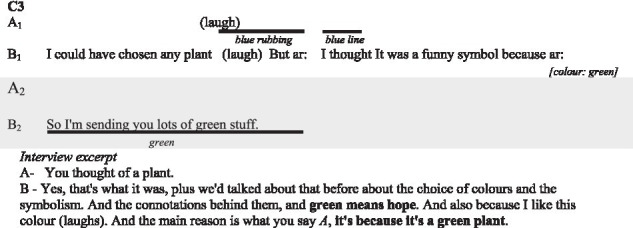


The interview excerpt of above illustrates how the participants try to associate colours to culturally accepted meanings (the participants looked at the significations of colours during the first break). Relying on culturally shared signification is however not always pertinent as colours often carries multiple significations. In the current matter, A only think about the relation to the plant and not to hope as B suggests.

The other partners of C3 and C4, explain being more spontaneous and driven by their liking of the colour. Their choices were guided by broad principles: bright, dark or pastel colours depending on the situation; without settling on a particular colour.


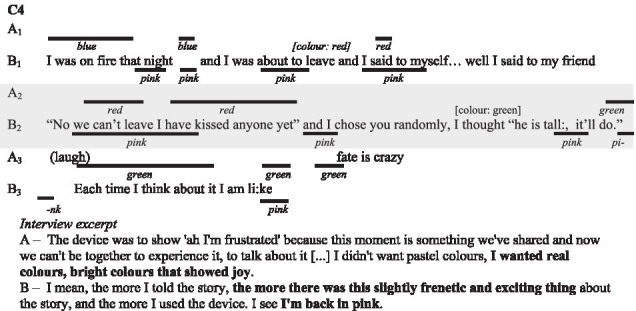


Colour is only accessible through the visual modality. However, three couples (C1, C3, C4) reported looking at the device very little and therefore not paying attention to the colour they received (even though, they were deliberate with their use of colour). This might explain why the colour choices when used outside of illustrative touches are rarely discussed.


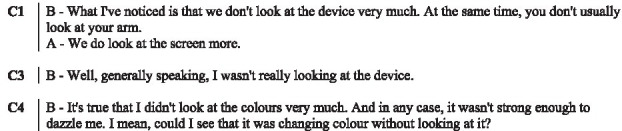


##### Illustrative aspects of visual-touches

Some participants produced more complex drawings. These illustrative aspects can directly participate in the construction of the meaning. Couples C1, C2 and C3 sought to reproduce the physical or conceptual characteristics of their story, as we saw above. Participants also mentioned drawing hearts to convey affection.


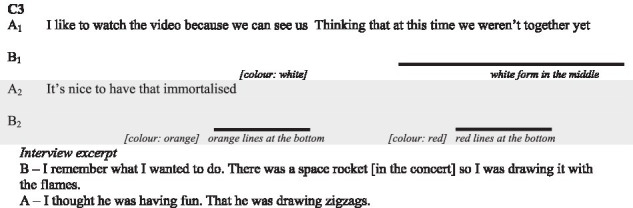


Most of the time the use of drawings is understood by the participants and contributes to the understanding of the visual-touch behaviours. In this case, however, the participant thinks her partner is only playing with the device.

#### 4.2.3 The verbal indicators

As the uses of visual-touch take place in a verbal interaction, it seems obvious that the verbal content will play a role in the elaboration of functions.

##### Explication

The simplest way for couples to understand their use of the device is to verbalise their action. It enables partners to pay closer attention to the stimuli, or specify the function they wish to associate with it. In some cases, this helps establish a code that can be reused later on.


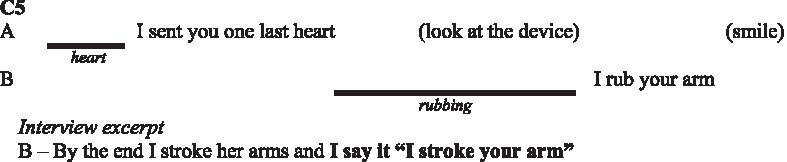


In the example, A looks at the device trying to understand what is happening. B then clarifies his action. The elaboration is completed with the smile of participant A, acknowledging her understanding.

Explications have been reported by all the couples to some extent (explication of the form, the function or both.)


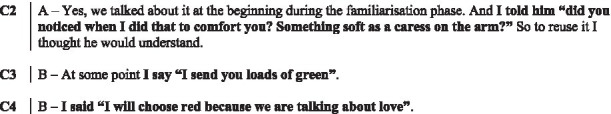


Looking at the “pauses” between protocol phases, we are able to highlight further aspects of explicit grounding. This could relate to the way they used the devices in general,


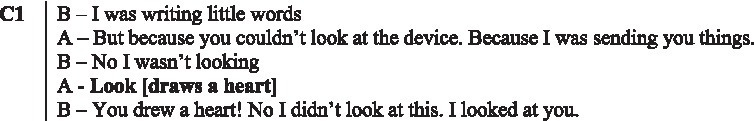


question their partner’s perception about the visual-touch stimuli,


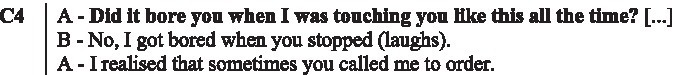


test the limits and possibilities of the system,


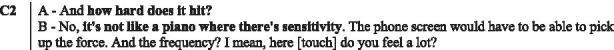


or, as we have just seen, explain a specific code.


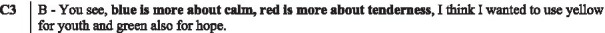


##### Theme and memory

Beyond mere explications, the verbal content linked to memory enables the couple to understand the functions (C1, C2, C3, C4). The importance of the theme of discussion is the most reported indicators (42 out of 71 behaviours). Touch can then act as an alert, allowing them to pay attention to the context at emotionally charged moments or to other para-verbal elements of the interaction that we present later. In this regard, as they are recalling shared events, prior knowledge of these events makes it easier for the couples to co-elaborate the functions.


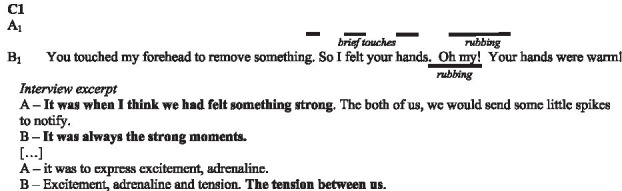


In this interview excerpt, participants are discussing the way visual-touch was used when they were talking about emotionally-charged events.

As most participant recall, the understanding of the visual-touch is tied to the context of the interaction. It helps place emphasis on what already lies in the context.


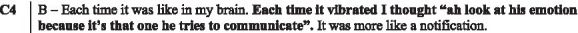


#### 4.2.4 Paraverbal indicators

Aside from what participants are discussing, several paraverbal indicators help the participants understand visual-touch.

##### Alignment and prosody

One of the participants in C2 wilfully aligned the rhythm of the visual-touch and the rhythm of his speech, so that his partner was attentive to the form and function.


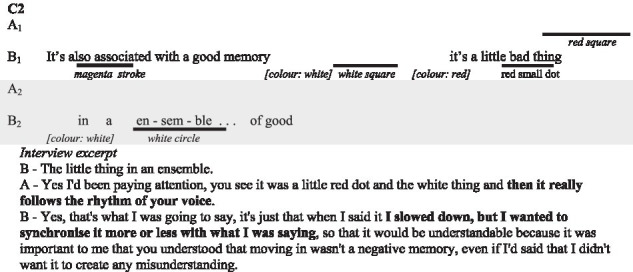


In this example, the participants specifically discuss the role played by the alignment between the prosody and the touch patterns of B in the understanding of the illustration. The other couples were not explicit about alignment and their prosody. Though, we are able to identify similar alignments within the reported visual-touch, especially with C1.


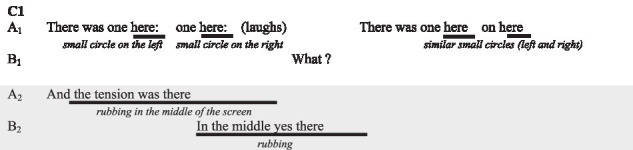


Here, we see how A synchronises her touch in space and time with the words “here.” With regards to the elaboration of meaning we also notice the repetition of this sequence, following B’s interrogation, as part of a grounding process. Theses alignments between the deictic words “here” and the small circles drawn by A, while not mentioned in interview, help for the understanding of the illustration functions.

Otherwise, the most common alignments are observed when the participants are laughing and using the device simultaneously, in a way serving emotional emphasis purposes.

##### Interaction: role and time

Participants evoke how they used the device differently whether they were speakers or listeners (C1, C3, C4, C5). This is often related to functions such as backchannels and adaptors.


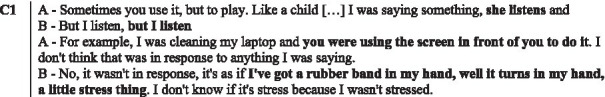


Another aspect of the interaction is the timing of the touch. In that regard, participants only evoke how touch was used as closure (C3 and C5).


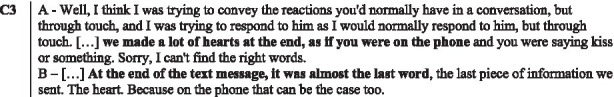


#### 4.2.5 Nonverbal indicators

To conclude, participants evoke the role played by nonverbal indicators, such as gaze direction and facial expression in the understanding of meaning.

##### Gaze and facial expression

The participants (C1, C4, C5) are particularly interested in the orientation of their partner’s gaze in order to determine the orientation of the touch (e.g., communication or adaptor). They also make the link between their partner’s facial expressions and the perceived touches.


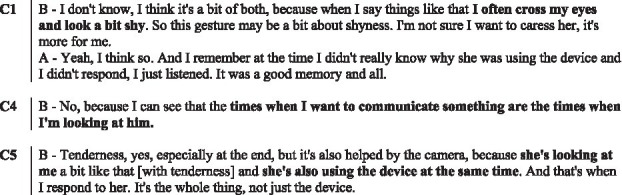


In these examples, we see how gaze and facial expression help, in association with other indicators such as the theme, to understand the functions.

##### Unconscious visual-touch mimicry

Finally, participants (C1, C2, C3, and C4) noted, often without being aware of it during the interaction, that they aligned their choice of colour, the rhythm, and the pattern of the touches. Not surprisingly mimicry was not limited to the tactile aspects of the visual-touches but also to their visual characteristics. In fact, we observe several cases where they used very similar colours without realising it before our interviews, or where the interactants produced the same type of movement, sometimes to the point of being identical.


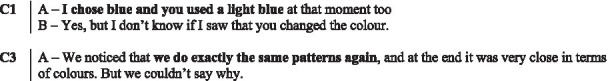


We assume that these alignments also helped to facilitate the sharing of representations between the interactants, as expressed by one of the participants in pair C2 regarding the similar use of illustrative elements they had at the beginning of the extract.


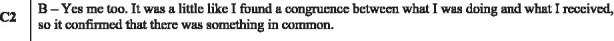


Here, the perception of congruent visual-touches played a role in the grounding processes.

### 4.3 Degree of mutual understanding (RQ3)

As mentioned in our analysis and noticed in the examples presented above, the interactants did not always understand each other. Even while discussing their exchange in interviews, they did not agree on every function of the produced and perceived visual-touches.

**Figure 5:**
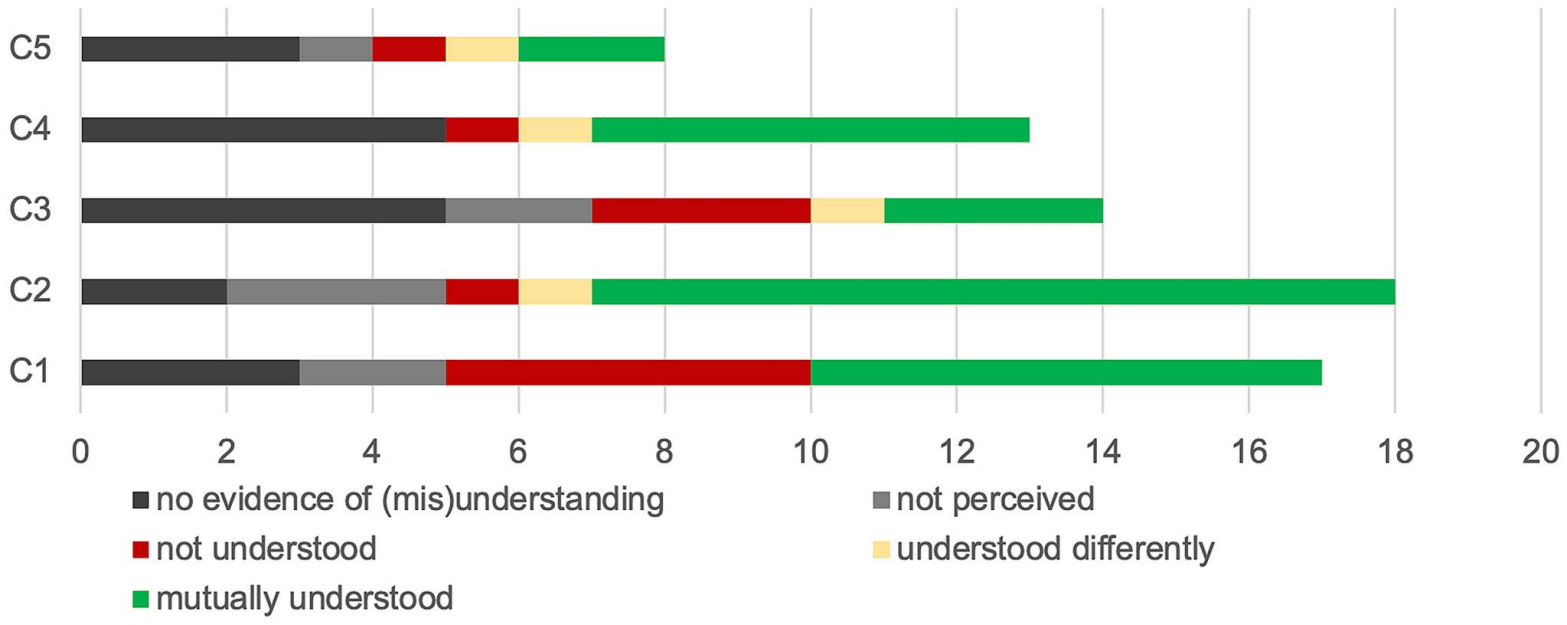
Mutual understanding of the visual-touches. This graph illustrates the mutual understanding levels. Each couple (C1 to C5) is represented on a line and from left to right is presented the number of visual-touch behaviours which are: discussed but no evidence of understanding or misunderstanding is accessible, not perceived by the receiver, not understood by the receiver, understood differently from the sender intention, or mutually understood.

For each reported visual-touch behaviour, we identified to what extent there was mutual understanding, on the basis of the interaction and of the interview. Figure 5 presents the different degree of mutual understanding: (1) no evidence,^1^ (2) the receiver did not perceive the visual-touch, (3) the receiver perceived the visual-touch but did not understand it, (4) the receiver perceived the visual-touch but understood it differently to the sender’s intent, and (5) the two interactants share the same understanding of the touch.

**Figure 6:**
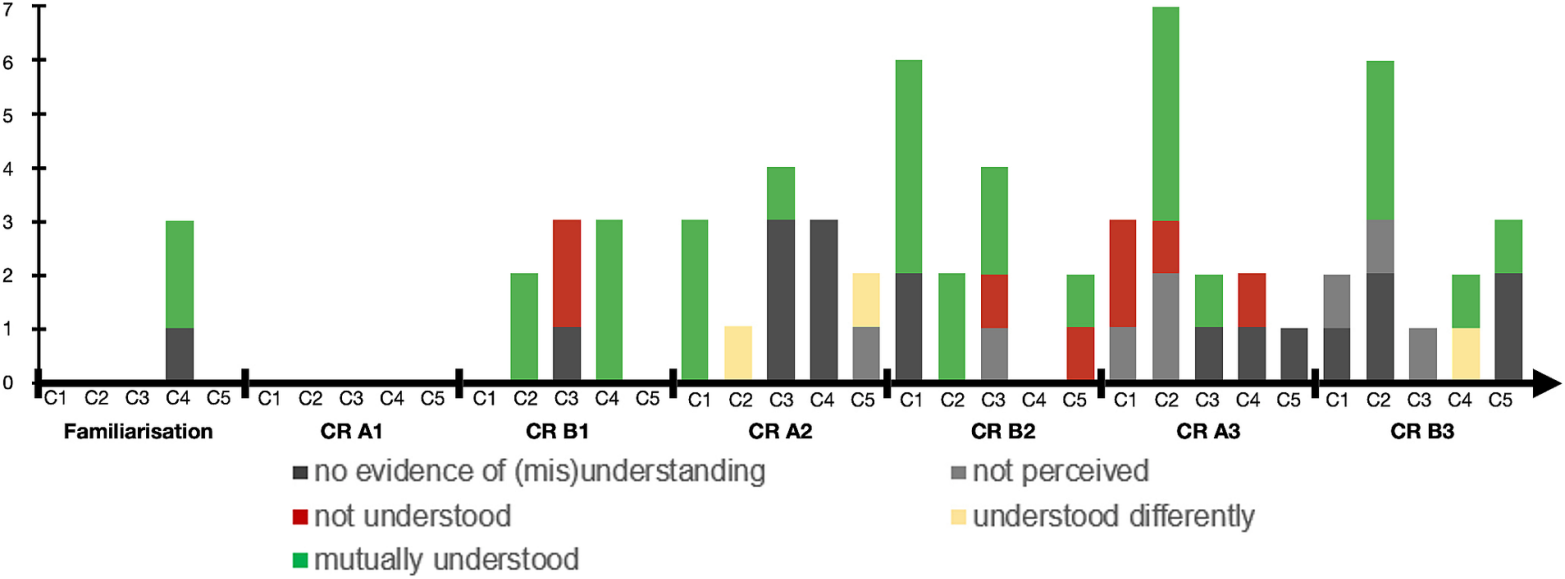
Mutual understanding evolution over the course of the interactions. The figure presents the mutual understanding of the visual-touch behaviours distributed across the interaction phases for each the couple (C1 to C5), from the familiarisation to the last memory of participants B (CR B3: Third memory of participants B in the Collaborative Remembering phase).

First, plotting the mutual understanding degrees on the timeline of the interactions, we hoped to observe higher degree of shared understanding by the end of the interaction, with regards to the construction of common ground and routinisation processes as suggested by our theoretical framework. However as presented in Figure 6, we see no such progression, which might be explained by the relatively short duration of the collaborative remembering phase.

Trying to correlate the degree of mutual understanding to the functions or the indicators reported by the participants, we first notice that the most understood visual-touch behaviours are those used to illustrate the related stories (‘illustration’) as well as those communicating ‘positive affects’. Both these functions are also the most reported, hence we can assume that participants tend to mention moments when they think they understood each other better.

In a similar manner, some indicators are more often associated with mutual understanding. *Theme* and *Colours* show the higher number of reported indicators associated with mutually understood behaviours, however those are also the two most reported indicators. *Explication* and *Illustrative aspects* on the other hand, not reported as much, are highly associated to mutual understanding.

As the co-elaboration process is multifactorial, the indicators are used as a body of cues helping in the elaboration of the functions. Hence, we assume that the more indicators considered, the better the mutual understanding. For instance, the three couples who understood each other best (C1, C2 and C4) were also those who reported using the greatest number of elements (cf. Table 4; Figure 5).

## 5 Discussion

Following an interactionist approach to study how visual-touch is used in distant verbal interactions, we are able to answer two of our research questions related to the specificity of visual-touch in the elaboration of the functions (RQ1) and to the indicators considered by interactants to attribute functions to visual-touch (RQ2). We are also partially able to answer our question related to the extent of mutual understanding (RQ3).

The three following subsections (5.1, 5.2, and 5.3) discuss our results related to these questions.

### 5.1 The specificity of visual-touch functions (RQ1)

Our results highlight 13 functions of visual-touch, 11 of which were previously identified by Héron et al. (2022). Here we go a step further by classifying these uses of the VisualTouch device with regards to dimensions of functions of verbal interaction (Vion, 1992): *interaction management*, *meaning making*, and *relationship building;* and by investigating the visual aspects of this specific case of mediated social touch.

A specificity of mediated touch and more specifically of visual-touch is that we observe a redistribution of the functions over interaction modalities compared to actual touch. In the following subsections, we discuss each dimension of functions and the observed redistributions.

#### 5.1.1 Interaction management

Only a few instances of *backchannel* and *turn-giving* are reported in our results. These behaviours—which can be explained as the low-level automatic processes mentioned in the interactive alignment model (Garrod and Pickering, 2009)—allow for fluid dialogues and are very often subconsciously accomplished. Hence, it is not surprising that our participants did not mention many functions of this category, as previously observed by Héron et al. (2022).

In face to face interaction, these functions are mostly supported by other para- and nonverbal behaviours such as glances and co-verbal gestures (Jokinen et al., 2010; Wagner et al., 2014) or pointing gestures (Mondada, 2004).

#### 5.1.2 Relationship building and maintenance

In our study, we identify functions that have already been reported (for both mediated and actual touch) such as the communication of *positive* and *negative affects* (Hertenstein et al., 2006, 2009; Huisman, 2017; Jones and Yarbrough, 1985; van Erp and Toet, 2015). In addition, while we did not evaluate the feeling of presence, we observed a *play* function (where participants are having fun with the device) sometimes called *mimicry* (e.g., Chang et al., 2002) which we believe helps the feeling of connectedness. In our work, mimicry was reported and observed with the visual-touch rhythm, patterns, as well as in the choice of colours. Research indicates that mimicry is closely related to social influence (Bailenson and Yee, 2005) and group membership (Bourgeois and Hess, 2008).

#### 5.1.3 Meaning-making

Our results indicate that visual-touches support many functions associated with the interpretation of meaning previously highlighted in the literature. *Emphasis*, which allows participants to highlight certain parts of their utterances, was also observed by Chang et al. (2002) for mediated touch. The functions of *modulation*—to alter the tone of utterances—*referring*—for the interactants to clarify the objects of the exchange—have already been partially highlighted in face-to-face interaction by Jones and Yarbrough (1985) with “playful touch” and “reference to appearance,” respectivly. Our results highlight a new function of mediated-touch, *illustration*, which occurrs through iconic gestures in face-to-face interactions (McNeill, 1992). This transfer of functions is not limited to *illustration*. For instance, in co-presence, *emphasis* is mostly supported by co-verbal gestures (Bull and Connelly, 1985; Jokinen et al., 2010; Wagner et al., 2014) and intonations (Arons, 1994; Ladd and Morton, 1997), while *referring* is mostly achieved through deictic gestures (Lefebvre, 2008), and *modulation* with prosodic elements (e.g., ironic tone).

#### 5.1.4 A redistribution of the functions over modalities

The several transfers of functions to visual-touch that we observe in mediated communication could have at least three explanations. First, the limitations of video calls which do not necessarily guarantee a good understanding of who is speaking (e.g., overlap) and what is being referred to, create difficulty in identifying the orientation of gazes and co-verbal gestures (Bitti and Garotti, 2011; Olson and Olson, 2000). In mediated contexts, gestures are reduced (Lefebvre, 2008). Indeed, in our study participants did not mention co-verbal gestures. Looking at the video, participants did not produce many gestures and when they did, they were mostly not visible through the webcam. In addition, potential discomfort associated with wearing the device could explain the limited gestures (two participants talked about the weight of the device and one indicated that the device was sometimes limiting in terms of movements). Second, the differences of properties between actual and visual-touch could explain these transfers of functions, as the specificity of visual-touch is the visual dimension enabling people to illustrate physical features, emotions, or other concepts, related to their current narrative. Third, the mere participation in the experiment could also contribute to increased use of VisualTouch. While we aimed at naturalistic interactions, knowing the aim of the study (explore the use of a mediated touch device), as well as the novelty effect, could explain participants’ dedication to use the device thoroughly.

### 5.2 The visual-touch characteristics in the determination of functions (RQ1 and RQ2)

While the visual modality was originally thought to increase the perceived accuracy of the tactile stimuli, our results indicate some form of autonomy. Participants talk about the colour they send, as if the tactile aspects were not present: “I send you some green.” They also report using a colour code and drawing (not just for illustration) precise forms. Research suggests that the frequent occurrence of complex iconic gestures and numerous pointing gestures are associated with the introduction of novel components of the discourse (Levy and McNeill, 1992; Parrill, 2010), hence we can assume that they are closely related to the construction of common ground. Indeed, there are accounts of the cross-modal relationship between the semantic and gestural modalities in speech coordination (Rasenberg et al., 2022) and the priming effect (Yap et al., 2011) for instance. We can say that the illustrative aspects of the visual-touch behaviours actively participate in the elaboration of the understanding of the functions. However, from the receiver perspective, participants did not seem to always pay much attention to visual aspects and were more concerned about the cadence, the rhythm of the touch.

Besides illustrative aspects of visual touch, the choice of colour was largely commented on by our participants, with results diverging from what laboratory studies report. In the literature, blue is often associated with positive affects and red with negative affects (Suk and Irtel, 2008; Valdez and Mehrabian, 1994; Wilms and Oberfeld, 2018; Zhang et al., 2019). Our results show that red can also be positive when associated with love and passion, while blue can be linked to sadness. Similarly, the relation between touch forms and their valence will vary depending on situations. Results from a limited context setting should be taken with caution, as recent research on actual touch suggests that the physical characteristics play little role in the determination of positive touch experience (Sailer et al., 2024).

As hypothesised in our framework, we consider that the context is responsible for meaning shifts. Our study is concerned with the functions of mediated touch in social interaction, so it is not surprising that a meaning associated with a stimulus with no context is re-evaluated during the interaction in relation to the context. In short, apart from the illustration function, the visual aspects do not matter much, as also suggested by our results.

These observations could lead to rethinking the design of the VisualTouch device. Previous research indicates that congruent visual-touch patterns (i.e., identical for visual and tactile cues) lead to a wider emotional communication in comparison to tactile cues alone (Zhang et al., 2019). However, here we note that in interaction, participants do not often take into account the visual pattern as they are looking at their partner, and that the perception of the cadence and broad patterns of the touch and colours are sufficient in combination with the interactional context and the knowledge of shared memories.

### 5.3 The role of the interactional context (RQ2)

Several studies point out the critical role of the context for social touch functions, in co-present (Jones and Yarbrough, 1985) or distant/mediated interactions (Price et al., 2022). Our results help to understand how participants attribute meanings to the visual-touch they produce and receive by considering the interactional context. Not surprisingly, the indicators reported by the participants comprise verbal, para-verbal and non-verbal behaviours, as the context is key to determining the relation between forms and meanings (Rasenberg et al., 2020). Our results reaffirm the ability of *grounding* and *interactive alignment* theories to highlight the explicit and implicit negotiation processes deployed by interactants to co-construct mediated-touch functions in interaction.

#### 5.3.1 The verbal material

The theme of the sequences and the participants’ shared knowledge of memories contribute to the construction of functions by drawing on their common ground. Price et al. (2022) notably highlight that close friends, family and partners developed idiosyncratic meanings on associations with the touch characteristics and their shared touch histories. The common knowledge of their relationship is key for both the sender and receiver. The interactants also make explicit their use of the device. They announce the sending of certain colours and lines or specify the function they attribute to them. This directly echoes the principle of least collaborative effort set out in *grounding* theory: from a collaborative point of view, it is easier to provide clarification when a statement seems unclear rather than waiting for the interactant to ask for clarification (Clark and Brennan, 1991; Clark and Wilkes-Gibbs, 1986). Sometimes this is done before or after the repeated use of a form of touch. We also note the use of pauses to discuss the use of the device, understand their physical possibilities and determine codes.

#### 5.3.2 The para- and non-verbal material

Automatic or conscious intermodal alignments further support the link between the verbal modality and the occurrence of visual-touch. Interestingly, we observed a mostly unconscious process being used deliberately by a participant to emphasise verbal content. From an interactive alignment perspective, these oftentimes unconscious alignments play a role in the forthcoming mutual understanding. The literature also reports alignments both within and across modalities (e.g., speech or gestures) and amongst interactants (Kimbara, 2008; Louwerse et al., 2012; Tabensky, 2001; Holler and Wilkin, 2011) in line with the interactive alignment model suggesting the interrelation between modalities (Pickering and Garrod, 2004). More importantly behavioural alignments are associated with successful communications (Fay et al., 2018) and gestural co-construction (Oben and Brône, 2016).

Non-verbal behaviours such as facial expressions also play a part in the construction of functions, as do the glances that allow us to understand the orientation of the touches (i.e., communicative or adaptive functions). Participants also report alignments in colour choices and in certain touch shape characteristics, although they are not always aware of them. It is likely that these alignments promote the understanding of touches and therefore participate in the co-elaboration of meaning.

### 5.4 The co-elaboration process and mutual understanding (RQ3)

Park et al. (2013) showed that over a long period of time participants stabilised the form-function relation for a few meanings as would be the case for idiomatic expressions.

In our studies, participants came up with various functions for the device. The visual-touch forms and their associated functions were context-specific and each couple relied upon different characteristics of the stimuli (colour, pattern, duration, rhythm, etc.) to co-elaborate meaning. For instance, participant A of C2 explicitly associated the context (something sad or difficult) and the touch-form (a slow white stroke) with a function of comforting, which she re-used in the interaction. This can be explained from the point of view of common ground (Clark and Schaefer, 1989), as well as the concept of routinisation proposed by the interactive alignment model (Pickering and Garrod, 2004). Over the course of the interaction, co-activations of different forms and functions of touch, and processes of explicit negotiation, can eventually lead to the formation of more durable form-function associations, i.e., the construction of a specific common frame of reference for each couple.

The consideration given to the different indicators and the dynamics of the interaction inevitably leads to different degrees of mutual understanding. Our results in that regard are not completely conclusive. On the one hand, certain indicators, such as Theme, Colours, Illustrative aspects, and Explication, are associated with a higher degree of mutual understanding. On the other hand, it seems that participants who mentioned more indicators of visual-touch characteristics, the verbal, para- and non-verbal context, better understand each other. Throughout the presentation of our results, we specified, when possible, how mutual understanding was achieved. What stands out is the dynamic and factorial aspect of the co-elaboration process regarding the indicators reported. They are combined to participate in the construction of meaning.

Our results report several cases of misunderstanding or incomplete understanding. In those cases, we could consider that the interactants reached a common ground sufficient for the continuation of the interaction but did not fully reach mutual understanding. As Cherubini et al. (2005) pointed out, grounding at the utterance level is not equivalent to mutual understanding. Drawing from the cognitive environment concept (Wilson and Sperber, 2006), they stress that it is not because common ground is achieved that people share understanding, only that they are able to do so.

In the interviews, we noted cases where participants tried to rely on what they believed to be culturally shared meanings (be it for colours or patterns). These are instances of perspective taking—participants “considering how a given utterance would be likely to be interpreted by the receiver” (Micklos and Woensdregt, 2023). Though relying on cultural aspects was not always successful because of the nonunivocal relation between form and function, and interactants associated different meanings to the visual-touches. From the receiver perspective, the assumed intention of the toucher or the purpose of the touch received plays an important role in how it is interpreted (Sailer and Leknes, 2022; Sailer et al., 2024). Relying on the already developed common ground appears to be one of the most important factors for the successful achievement of the co-elaboration of meaning, as suggested by our results, participants frequently mentioning the importance of theme and shared knowledge about the memory. Oben and Brône (2016), for instance, emphasise the importance of the historical perspective of grounding as their results suggest that lexical and gestural alignments cannot solely be explained by interactive alignment. The relationship of our participants might have played a crucial role in mutual understanding. We indeed observe higher degree of mutual understanding for participants reporting more consideration of the Theme indicator—which is associated with the theme of the story and its shared knowledge.

## 6 Limitations

Even though our work aimed to highlight the functions of the visual-touches and the indicators used by the interactants to co-elaborate these functions was mostly achieved, some limitations are worth noting, on the basis of which we can propose perspectives for future research.

One limitation is the participant selection. We developed a complex protocol for in-depth analysis and selected a limited number of participants, making generalisation difficult. Additionally, we focused exclusively on cohabiting couples to observe a wide range of visual-touch behaviours, which restricted diversity of our population. Whereas previous research indicates the importance of relationship quality on the perception of social touch (Jakubiak, 2022; Sailer et al., 2024), we only recorded the duration (3 to 6 years) of relationships, without assessing their quality. While our aim was to explore the interactional context factors influencing the functions of visual-touch, evaluating the quality of relationships could have provided valuable insights. Future research should consider a more diverse participant pool in terms of relationship types, age, professional backgrounds, living environments (as all participants were upper-middle-class residents near Paris), and digital literacy (which we did not assess in this study).

Another limitation is the study’s time frame. Our interactionist approach emphasises that meaning is collaboratively constructed over time, yet the brief nature of the interactions in our study did not allow us to evaluate long-term negotiation processes. We plan to conduct a longitudinal study with a broader participant base to better understand visual-touch in technology-mediated interactions.

To conclude, we noted limitations related to the devices used for visual-touch. Social touch is a complex phenomenon that consists of “more than tactile stimulation alone, and is accompanied by a rich set of multimodal cues” (p.15, Ipakchian Askari et al., 2022). Thus, the design of the device may influence interactant behaviours, as evidenced by the frequent use of illustrations and colours in our study. Moreover, we observed minor delays in touch stimuli transmission and video calls, though participants did not report any noticeable delays. Future research should explore the impact of these delays on meaning co-elaboration, particularly as participants navigate the interactional context for mutual understanding.

## 7 Conclusion

In our study, five couples each interacted during a 1-h session at their homes. The interactions were realised though video call with the addition of a visual-touch device enabling participants to enrich their communication. We conducted confrontation interviews with each couple using the video recordings of their interactions. We investigated the functions associated to the visual-touches and the processes by which these meanings were created and understood. Our aim was to understand *how the functions of visual-touch emerge in social interaction.*

With our results we are able to answer our first research question regarding the specificity of visual-touch in interaction (RQ1) as we show that (1) visual-touch enables a variety of functions with some specific to the visual aspect of the device and (2) that these functions are redistributed from the modalities of communication onto the visual touch channel. We also described (3) the verbal, paraverbal and non-verbal context indicators considered by the interactants to elaborate the functions of visual-touches, thus answering our second research question (RQ2). We partially answer our third research question (RQ3) by highlighting the (4) multifactorial and dynamic aspects of the co-elaboration process, meaning that the presence of indicators and grounding do not always lead to mutual understanding. Nevertheless, we lack a complete understanding of this dynamic process, which we wish to explore further in a future study through the fine-grained analysis of the interactions. Contrary to what was expected with our theoretical framework, we did not observe increasingly higher degree of mutual understanding over time. However, with common ground building and routinization in relation with interactive alignments we should observe more convergence in the relation between forms and meanings (Galantucci et al., 2012; Oben and Brône, 2016; Fay et al., 2018) and therefore a higher degree of mutual understanding. This could be explained by the short duration of the collaborative remembering phase (1 hour) and its varied thematic context, which did not give the opportunity to the participants to converge on routinised form and function relationships consistently. In the future, we wish to explore the use of visual-touch in naturalistic settings over longer periods of time.

Our study offers a twofold original contribution. Firstly, we highlighted an Illustration function in mediated touch, akin to what is seen in co-speech gestures during face-to-face interactions, which has not been previously associated with touch. Secondly, we demonstrated the interconnectedness of cultural and idiosyncratic contextual meanings in the context of mediated touch, reinforcing the importance of considering context when conducting research on touch, whether mediated or direct.

## Data Availability

The raw data supporting the conclusions of this article will be made available by the authors, without undue reservation.

## Footnotes

^1^In 18 cases, there is no clear evidence of mutual understanding or misunderstanding, neither in the interaction itself or in the interview.

## References

[ref1] Alea N. Bluck S. (2003). Why are you telling me that? A conceptual model of the social function of autobiographical memory. Memory 11, 165–178. doi: 10.1080/741938207, PMID: 12820829

[ref2] Alsamarei A. A. Şener B. (2023). Remote social touch framework: A way to communicate physical interactions across long distances. J. Multimodal User Interfaces 17, 79–104. doi: 10.1007/s12193-023-00402-z

[ref3] Arons B. (1994). “Pitch-based emphasis detection for segmenting speech recordings.” in *Proceedings of Third International Conference of Spoken Language Processing*. pp. 1931–1934.

[ref4] Attardo S. Eisterhold J. Hay J. Poggi I. (2003). Multimodal markers of irony and sarcasm. Int. J. Humor Res. 16:12. doi: 10.1515/humr.2003.012

[ref5] Bailenson J. N. Yee N. (2005). Digital chameleons: automatic assimilation of nonverbal gestures in immersive virtual environments. Psychol. Sci. 16, 814–819. doi: 10.1111/j.1467-9280.2005.01619.x, PMID: 16181445

[ref6] Bailenson J. N. Yee N. Brave S. Merget D. Koslow D. (2007). Virtual interpersonal touch: expressing and recognizing emotions through haptic devices. Hum. Comp. Interact. 22, 325–353. doi: 10.1080/07370020701493509

[ref7] Baker M. J. (2004). Recherches Sur l’élaboration de connaissances dans le dialogue [research on the development of knowledge in dialogue]. [Habilitation à diriger la recherche]. [Nancy (FR)]: Université de Nancy II. <tel-00110314>.

[ref8] Banse R. Scherer K. R. (1996). Acoustic profiles in vocal emotion expression. J. Pers. Soc. Psychol. 70, 614–636. doi: 10.1037/0022-3514.70.3.614, PMID: 8851745

[ref9] Bietti L. M. Baker M. J. Détienne F. (2016). Joint remembering in collaborative design: A multimodal approach in the case of a video design studio. CoDesign 12, 221–242. doi: 10.1080/15710882.2015.1103752

[ref10] Bietti L. M. Galiana Castelló F. (2013). Embodied reminders in family interactions: multimodal collaboration in remembering activities. Discourse Stud. 15, 665–686. doi: 10.1177/1461445613490010

[ref11] Bitti P. E. R. Garotti P. L. (2011). “Nonverbal communication and cultural differences: issues for face-to-face communication over the internet” in Face-to-face communication over the internet. eds. Kappas A. Kramer N. C. (Cambridge: Cambridge University Press), 81–99.

[ref12] Bourgeois P. Hess U. (2008). The impact of social context on mimicry. Biol. Psychol. 77, 343–352. doi: 10.1016/j.biopsycho.2007.11.008, PMID: 18164534

[ref13] Brône G. Zima E. (2014). Towards a dialogic construction grammar: ad hoc routines and resonance activation. Cognit. Linguist. 25, 457–495. doi: 10.1515/cog-2014-0027

[ref14] Bryant G. A. Fox Tree J. E. (2005). Is there an ironic tone of voice? Lang. Speech 48, 257–277. doi: 10.1177/00238309050480030101, PMID: 16416937

[ref15] Buck R. Losow J. I. Murphy M. M. Costanzo P. (1992). Social facilitation and inhibition of emotional expression and communication. J. Pers. Soc. Psychol. 63, 962–968. doi: 10.1037/0022-3514.63.6.962, PMID: 1460562

[ref16] Bull P. Connelly G. (1985). Body movement and emphasis in speech. J. Nonverbal Behav. 9, 169–187. doi: 10.1007/BF01000738, PMID: 39399483

[ref17] Bunt H. C. (1994). Context and dialogue control. THINK Quart. 3, 19–31.

[ref18] Burgoon J. K. Guerrero L. K. Floyd K. (2016). Nonverbal communication. New York: Routledge.

[ref19] Cabibihan J.-J. Zheng L. Cher C. K. T. (2012). “Affective tele-touch” in Social robotics. eds. Ge S. S. Khatib O. Cabibihan J.-J. Simmons R. Williams M.-A., vol. 7621 (Berlin Heidelberg: Springer), 348–356.

[ref20] Cahour B. Licoppe C. Créno L. (2018). Articulation fine des données vidéo et des entretiens d’auto-confrontation explicitante: Étude de cas d’interactions en covoiturage [fine-grained articulation of video data and of explicating self-confrontation interviews: a case study of carpooling interactions]. Le travail humain 81, 269–305. doi: 10.3917/th.814.0269

[ref21] Cahour B. Salembier P. Zouinar M. (2016). Analyzing lived experience of activity. Le travail humain 79, 259–284. doi: 10.3917/th.793.0259, PMID: 39517377

[ref22] Chang A. O’Modhrain S. Jacob R. Gunther E. Ishii H. (2002). “ComTouch: design of a vibrotactile communication device”. in *Proceedings of the Conference on Designing Interactive Systems Processes, Practices, Methods, and Techniques (DIS ‘02)*. pp. 312–320.

[ref23] Cherubini M. van Der Pol J. Dillenbourg P. (2005). “Grounding is not shared understanding: distinguishing grounding at an utterance and knowledge level”. in The fifth international and interdisciplinary conference on modeling and using CONTEXT (CONTEXT'05). <hal-00190089>.

[ref24] Chieffi S. Ricci M. (2005). Gesture production and text structure. Percept. Mot. Skills 101, 435–439. doi: 10.2466/pms.101.2.435-439, PMID: 16383076

[ref25] Clark H. H. Brennan S. E. (1991). “Grounding in communication” in Perspectives on socially shared cognition. eds. Resnick L. Levine J. Teasley S. (Washington, DC: American Psychological Association), 127–149.

[ref26] Clark H. H. Schaefer E. F. (1989). Contributing to discourse. Cogn. Sci. 13, 259–294. doi: 10.1207/s15516709cog1302_7, PMID: 39536473

[ref27] Clark H. H. Wilkes-Gibbs D. (1986). Referring as a collaborative process. Cognition 22, 1–39. doi: 10.1016/0010-0277(86)90010-7, PMID: 3709088

[ref29] Desnoyers-Stewart J. Stepanova E. R. Liu P. Kitson A. Pennefather P. P. Ryzhov V.. (2023). “Embodied telepresent connection (ETC): exploring virtual social touch through pseudohaptics”. in *CHI ‘23 Extended Abstracts on Human Factors in Computing Systems, 97*. pp. 1–7.

[ref30] Ditzen B. Neumann I. D. Bodenmann G. (2007). Effects of different kinds of couple interaction on cortisol and heart rate responses to stress in women. Psychoneuroendocrinology 32, 565–574. doi: 10.1016/j.psyneuen.2007.03.011, PMID: 17499441

[ref31] Duncan S. (1972). Some signals and rules for taking speaking turns in conversations. J. Pers. Soc. Psychol. 23, 283–292. doi: 10.1037/h0033031, PMID: 25814968

[ref32] Eads J. Moseley G. L. Hillier S. (2015). Non-informative vision enhances tactile acuity: a systematic review and meta-analysis. Neuropsychologia 75, 179–185. doi: 10.1016/j.neuropsychologia.2015.06.006, PMID: 26071257

[ref33] Ekman P. Friesen W. V. Ellsworth P. (1972). Emotion in the human face. New York: Pergamon Press inc.

[ref34] ELAN (2022). Max Planck Institute for Psycholinguistics, The Language Archive, Nijmegen, NL. (Version 6.3) [Computer software]. Available at: https://archive.mpi.nl/tla/elan/ (Accessed May 4th, 2022)

[ref35] Fay N. Walker B. Swoboda N. Garrod S. (2018). How to create shared symbols. Cogn. Sci. 42, 241–269. doi: 10.1111/cogs.12600, PMID: 29457653

[ref9001] Field T. Poling S. Mines S. Bendell D. Veazey C. (2020). Touch deprivation and exercise during the COVID-19 lockdown april. Medical Research Archives, 8:8. doi: 10.18103/mra.v8i8.2204

[ref36] Floyd K. (2014). Relational and health correlates of affection deprivation. West. J. Commun. 78, 383–403. doi: 10.1080/10570314.2014.927071, PMID: 20462422

[ref37] Floyd K. Melcher C. Zhong M. (2000). “Exceptional ways to end conversations: A cognitive-skills approach to communication” in Let’s talk: A cognitive-skills approach to interpersonal communication. eds. Waugh C. G. Gorden W. I. Golden K. M. (Newark (DE): Kendall/Hunt), 327–329.

[ref38] Galantucci B. Garrod S. Roberts G. (2012). Experimental semiotics. Language and linguistics. Compass 6, 477–493. doi: 10.1002/lnc3.351

[ref39] Gallace A. Spence C. (2010). The science of interpersonal touch: an overview. Neurosci. Biobehav. Rev. 34, 246–259. doi: 10.1016/j.neubiorev.2008.10.004, PMID: 18992276

[ref40] Garrod S. Pickering M. J. (2009). Joint action, interactive alignment, and dialog. Top. Cogn. Sci. 1, 292–304. doi: 10.1111/j.1756-8765.2009.01020.x, PMID: 25164934

[ref41] Giannopoulos E. Eslava V. Oyarzabal M. Hierro T. González L. Ferre M.. (2008). “The effect of haptic feedback on basic social interaction within shared virtual environments” in Haptics: Perception, devices and scenarios. ed. Ferre M., vol. 5024 (Berlin Heidelberg: Springer), 301–307.

[ref42] Goffman E. (1959). The presentation of self in everyday life. New York, NY: Bantam Doubleday Dell Publishing Group.

[ref43] Grewen K. M. Anderson B. J. Girdler S. S. Light K. C. (2003). Warm partner contact is related to lower cardiovascular reactivity. Behav. Med. 29, 123–130. doi: 10.1080/08964280309596065, PMID: 15206831

[ref44] Haans A. IJsselsteijn W. A. (2009). The virtual Midas touch: helping behavior after a mediated social touch. IEEE Trans. Haptics 2, 136–140. doi: 10.1109/TOH.2009.20, PMID: 27788077

[ref45] Hadar U. Butterworth B. (1997). Iconic gestures, imagery, and word retrieval in speech. Semiotica 115, 147–172. doi: 10.1515/semi.1997.115.1-2.147

[ref46] Henricson M. Ersson A. Maatta S. Segesten K. Berglund A.-L. (2008). The outcome of tactile touch on stress parameters in intensive care: a randomized controlled trial. Complement. Ther. Clin. Pract. 14, 244–254. doi: 10.1016/j.ctcp.2008.03.003 18940711

[ref9002] Hartcher-O’Brien J. Gallace A. Krings B. Koppen C. Spence C. (2008). When vision ‘extinguishes’ touch in neurologically-normal people: extending the Colavita visual dominance effect. Experimental brain research, 186, 643–658. doi: 10.1007/s00221-008-1272-5 18301885

[ref47] Héron R. Safin S. Baker M. Détienne F. (2022). “The functions of Computer-mediated touch at a distance: an interactionist approach” in Proceedings of the 21st congress of the international ergonomics association (IEA 2021), lecture notes in networks and systems. eds. Black N. L. Neumann W. P. Noy I., vol. 223 (Cham: Springer), 45–53.

[ref48] Heslin R. Boss D. (1980). Nonverbal intimacy in airport arrival and departure. Personal. Soc. Psychol. Bull. 6, 248–252. doi: 10.1177/014616728062010

[ref49] Holler J. Wilkin K. (2011). Co-speech gesture mimicry in the process of collaborative referring during face-to-face dialogue. J. Nonverbal Behav. 35, 133–153. doi: 10.1007/s10919-011-0105-6

[ref9003] Huisman G. (2017). Social touch technology: A survey of haptic technology for social touch. IEEE transactions on haptics, 10, 391–408. doi: 10.1109/TOH.2017.2650221 28092577

[ref50] Huisman G. (2022). An interaction theory account of (mediated) social touch. Front. Psychol. 13:830193. doi: 10.3389/fpsyg.2022.830193, PMID: 35592150 PMC9110885

[ref51] Huisman G. Darriba Frederiks A. (2013). “Towards tactile expressions of emotion through mediated touch”. in *CHI ‘13 Extended Abstracts on Human Factors in Computing Systems*. 1575–1580.

[ref90011] Hertenstein M. J. Keltner D. App B. Bulleit B. A. Jaskolka A. R. (2006). Touch communicates distinct emotions. Emotion. 6:528–533. doi: 10.1037/1528-3542.6.3.528 16938094

[ref90012] Hertenstein M. J. Holmes R. McCullough M. Keltner D. (2009). The communication of emotion via touch. Emotion. 9:566–573. doi: 10.1037/a0016108 19653781

[ref52] Ipakchian Askari S. Haans A. Bos P. Eggink M. Lu E. M. Kwong F.. (2020). “Context matters: the effect of textual tone on the evaluation of mediated social touch” in Haptics: Science, technology, applications. eds. Nisky I. Hartcher-O’Brien J. Wiertlewski M. Smeets J., vol. 12272 (Cham: Springer), 131–139.

[ref54] Ipakchian Askari S. Haans A. IJsselsteijn W. A. (2022). Uncovering terra incognita in the AHD design space: A review of affective haptic devices. Front. Comp. Sci. 4:795772. doi: 10.3389/fcomp.2022.795772

[ref55] Jakubiak B. K. (2022). Affectionate touch in satisfying and dissatisfying romantic relationships. J. Soc. Pers. Relat. 39, 2287–2315. doi: 10.1177/02654075221077280

[ref56] Janta H. Cohen S. A. Williams A. M. (2015). Rethinking visiting friends and relatives mobilities. Popul. Space Place 21, 585–598. doi: 10.1002/psp.1914

[ref57] Jewitt C. Price S. Steimle J. Huisman G. Golmohammadi L. Pourjafarian N.. (2021). Manifesto for digital social touch in crisis. Front. Comp. Sci. 3:754050. doi: 10.3389/fcomp.2021.754050

[ref9007] Jones S. E. Yarbrough A. E. (1985). A naturalistic study of the meanings of touch. Communications Monographs, 52, 19–56. doi: 10.1080/03637758509376094

[ref58] Jokinen K. Nishida M. Yamamoto S. (2010). “On eye-gaze and turn-taking”. in *Proceedings of the 2010 Workshop on Eye Gaze in Intelligent Human Machine Interaction - EGIHMI ‘10*. 118–123.

[ref59] Kendon A. (1986). Some reasons for studying gesture. Semiotica 62, 3–28. doi: 10.1515/semi.1986.62.1-2.3

[ref60] Kendon A. (1990). Conducting interaction: Patterns of behavior in focused encounters. Cambridge: Cambridge University Press.

[ref61] Kerbrat-Orecchioni C. (1996). La conversation [Conversation]. Paris: Seuil.

[ref62] Kimbara I. (2008). Gesture form convergence in joint description. J. Nonverbal Behav. 32, 123–131. doi: 10.1007/s10919-007-0044-4

[ref63] Knapp M. L. (1978). Non-verbal communication in human interaction. New York: Holt, Rinehart & Winston.

[ref64] Knapp M. L. Hart R. P. Friedrich G. W. Shulman G. M. (1973). The rhetoric of goodbye: verbal and nonverbal correlates of human leave-taking. Speech Monographs 40, 182–198. doi: 10.1080/03637757309375796

[ref65] Ladd D. R. Morton R. (1997). The perception of intonational emphasis: continuous or categorical? J. Phon. 25, 313–342. doi: 10.1006/jpho.1997.0046, PMID: 39538286

[ref66] Lécuyer A. (2009). Simulating haptic feedback using vision: A survey of research and applications of pseudo-haptic feedback. Presence Teleop. Virt. 18, 39–53. doi: 10.1162/pres.18.1.39

[ref67] Lefebvre L. (2008). Les indicateurs non verbaux dans les interactions médiatisées [the nonverbal indicators in mediated interactions]. [PhD thesis] [Lorient (FR)]: Université de Bretagne-Sud. <tel-00350409>.

[ref68] Levy E. T. McNeill D. (1992). Speech, gesture, and discourse. Discourse Process. 15, 277–301. doi: 10.1080/01638539209544813, PMID: 39404854

[ref69] Louwerse M. M. Dale R. Bard E. G. Jeuniaux P. (2012). Behavior matching in multimodal communication is synchronized. Cogn. Sci. 36, 1404–1426. doi: 10.1111/j.1551-6709.2012.01269.x, PMID: 22984793

[ref70] Mancini F. Bauleo A. Cole J. Lui F. Porro C. A. Haggard P.. (2014). Whole-body mapping of spatial acuity for pain and touch. Ann. Neurol. 75, 917–924. doi: 10.1002/ana.24179, PMID: 24816757 PMC4143958

[ref71] Master S. L. Eisenberger N. I. Taylor S. E. Naliboff B. D. Shirinyan D. Lieberman M. D. (2009). A picture's worth: partner photographs reduce experimentally induced pain. Psychol. Sci. 20, 1316–1318. doi: 10.1111/j.1467-9280.2009.02444.x, PMID: 19788531

[ref72] Maswood R. Rasmussen A. S. Rajaram S. (2019). Collaborative remembering of emotional autobiographical memories: implications for emotion regulation and collective memory. J. Exp. Psychol. Gen. 148, 65–79. doi: 10.1037/xge0000468, PMID: 30211580

[ref73] McNeill D. (1992). Hand and mind: What gestures reveal about thought. Chicago: The University of Chicago Press.

[ref74] Micklos A. Woensdregt M. (2023). “Cognitive and interactive mechanisms for mutual understanding in conversation” in Oxford research encyclopedia of communication. ed. Nobit G. W. (Oxford, England: Oxford University Press).

[ref75] Mollo V. Falzon P. (2004). Auto- and Allo-confrontation as tools for reflective activities. Appl. Ergon. 35, 531–540. doi: 10.1016/j.apergo.2004.06.003, PMID: 15374760

[ref76] Mondada L. (2004). Temporalité, séquentialité et multimodalité au fondement de l’organisation de l’interaction [temporality, sequentiality and multimodality at the root of the organisation of interaction]. Cahiers de linguistique française 26, 269–292.

[ref77] Nakanishi H. Tanaka K. Wada Y. (2014). “Remote handshaking: touch enhances video-mediated social telepresence”. in *Proceedings of the SIGCHI Conference on Human Factors in Computing Systems*. pp. 2143–2152.

[ref78] Newport R. Rabb B. Jackson S. R. (2002). Noninformative vision improves haptic spatial perception. Curr. Biol. 12, 1661–1664. doi: 10.1016/S0960-9822(02)01178-8, PMID: 12361568

[ref79] Oben B. Brône G. (2016). Explaining interactive alignment: a multimodal and multifactorial account. J. Pragmat. 104, 32–51. doi: 10.1016/j.pragma.2016.07.002

[ref80] Olry-Louis I. (2011). Psychologie socio-cognitive de la communication: applications aux champs de l’éducation et du travail [socio-cognitive psychology of communication: applications to the fields of education and work]. [Habilitation à diriger la recherche]. [Nancy (FR)]:Université de Nancy II. <tel-00704513>.

[ref81] Olson G. M. Olson J. S. (2000). Distance Matters. Hum. Comp. Interact. 15, 139–178. doi: 10.1207/S15327051HCI1523_4

[ref82] Park Y.-W. Baek K.-M. Nam T.-J. (2013). “The roles of touch during phone conversations: long-distance couples’ use of POKE in their homes.” in *Proceedings of the SIGCHI Conference on Human Factors in Computing Systems*. pp. 1679–1688.

[ref83] Parrill F. (2010). “The hands are part of the package: gesture, common ground and information packaging” in Empirical and experimental methods in cognitive/functional research. eds. Newman J. Rice S. (Chicago: University of Chicago Press), 285–302.

[ref9004] Pickering M. J. Garrod S. (2004). Toward a mechanistic psychology of dialogue. Behavioral and brain sciences, 27: 169–190. doi: 10.1017/S0140525X04000056 15595235

[ref84] Price S. Bianchi-Berthouze N. Jewitt C. Yiannoutsou N. Fotopoulou K. Dajic S.. (2022). The making of meaning through dyadic haptic affective touch. ACM Trans. Comp. Hum. Interact. 29, 1–42. doi: 10.1145/3490494

[ref85] Quek F. McNeill D. Bryll R. Duncan S. Ma X.-F. Kirbas C.. (2002). Multimodal human discourse: gesture and speech. ACM Trans. Comp. Hum. Interact. 9, 171–193. doi: 10.1145/568513.568514, PMID: 38314574

[ref9005] Ramachandran V. S. Rogers-Ramachandran D. (1996). Synaesthesia in phantom limbs induced with mirrors. Proceedings of the Royal Society of London. Series B: Biological Sciences, 263:1369, 377–386. doi: 10.1098/rspb.1996.0058 8637922

[ref86] Rantala J. Salminen K. Raisamo R. Surakka V. (2013). Touch gestures in communicating emotional intention via vibrotactile stimulation. Int. J. Hum. Comp. Stud. 71, 679–690. doi: 10.1016/j.ijhcs.2013.02.004

[ref87] Rasenberg M. Özyürek A. Dingemanse M. (2020). Alignment in multimodal interaction: an integrative framework. Cogn. Sci. 44:e12911. doi: 10.1111/cogs.12911, PMID: 33124090 PMC7685147

[ref9006] Rasenberg M. Pouw W. Özyürek A. Dingemanse M. (2022). The multimodal nature of communicative efficiency in social interaction. Scientific Reports, 12, 19111. doi: 10.1038/s41598-022-22883-w 36351949 PMC9646718

[ref88] Ryan L. Klekowski Von Koppenfels A. Mulholland J. (2015). ‘The distance between us’: A comparative examination of the technical, spatial and temporal dimensions of the transnational social relationships of highly skilled migrants. Glob. Netw. 15, 198–216. doi: 10.1111/glob.12054

[ref89] Sailer U. Friedrich Y. Asgari F. Hassenzahl M. Croy I. (2024). Determinants for positive and negative experiences of interpersonal touch: context matters. Cognit. Emot. 38, 565–586. doi: 10.1080/02699931.2024.2311800, PMID: 38362744

[ref90] Sailer U. Leknes S. (2022). Meaning makes touch affective. Curr. Opin. Behav. Sci. 44:101099. doi: 10.1016/j.cobeha.2021.101099

[ref91] Sallnäs E.-L. (2010). “Haptic feedback increases perceived social presence”. In Kappers A. M. L. Erp J. B. F. van Bergmann Tiest W. M. Helm F. C. T. van der (Eds.), Haptics: Generating and perceiving tangible sensations vol. 6192 (Berlin: Springer), 178–185.

[ref9008] Simner J. Ludwig V. U. (2012). The color of touch: A case of tactile–visual synaesthesia. Neurocase, 18, 167–180. doi: 10.1080/13554794.2011.568503 21787247

[ref92] Smith J. MacLean K. (2007). Communicating emotion through a haptic link: design space and methodology. Int. J. Hum. Comp. Stud. 65, 376–387. doi: 10.1016/j.ijhcs.2006.11.006

[ref93] Suk H.-J. Irtel H. (2008). Emotional response to simple color stimuli. KANSEI Eng. Int. 7, 181–188. doi: 10.5057/kei.7.181, PMID: 38552832

[ref94] Sumioka H. Nakae A. Kanai R. Ishiguro H. (2013). Huggable communication medium decreases cortisol levels. Sci. Rep. 3:3034. doi: 10.1038/srep03034 24150186 PMC3805974

[ref95] Tabensky A. (2001). Gesture and speech rephrasings in conversation. Gesture 1, 213–235. doi: 10.1075/gest.1.2.07tab

[ref97] Teyssier M. Bailly G. Pelachaud C. Lecolinet E. (2020). Conveying emotions through device-initiated touch. IEEE Trans. Affect. Comput. 13, 1477–1488. doi: 10.1109/TAFFC.2020.3008693

[ref98] Theureau J. (2010). Les entretiens d’autoconfrontation et de remise en situation par les traces matérielles et le programme de recherche « cours d’action » [Self-confrontation and situational interviews using physical traces and the ‘course of action’ research programme]. Revue d’anthropologie des connaissances 4:287. doi: 10.3917/rac.010.0287

[ref99] Thomas P. A. Kim S. (2021). Lost touch? Implications of physical touch for physical health. J. Gerontol. 76, e111–e115. doi: 10.1093/geronb/gbaa134, PMID: 32845008 PMC7499739

[ref100] Traum D. R. Hinkelman E. A. (1992). Conversation acts in task-oriented spoken dialogue. Comput. Intell. 8, 575–599. doi: 10.1111/j.1467-8640.1992.tb00380.x

[ref101] Tsalamlal M. Y. Ouarti N. Martin J.-C. Ammi M. (2013). “EmotionAir: perception of emotions from air jet based tactile stimulation”. in *Humaine Association Conference on Affective Computing and Intelligent Interaction*. pp. 215–220.

[ref9009] van Erp JBF Toet A. (2015). Social touch in human–computer interaction. Front. Digit. Humanit. 2:2. doi: 10.3389/fdigh.2015.00002

[ref102] Valdez P. Mehrabian A. (1994). Effects of color on emotions. J. Exp. Psychol. Gen. 123, 394–409. doi: 10.1037/0096-3445.123.4.394, PMID: 7996122

[ref103] Vion R. (1992). La communication verbale [the verbal communication]. Paris: Hachette.

[ref104] Wagner P. Malisz Z. Kopp S. (2014). Gesture and speech in interaction: an overview. Speech Comm. 57, 209–232. doi: 10.1016/j.specom.2013.09.008, PMID: 39302887

[ref105] Whitcher S. J. Fisher J. D. (1979). Multidimensional reaction to therapeutic touch in a hospital setting. J. Pers. Soc. Psychol. 37, 87–96. doi: 10.1037/0022-3514.37.1.87, PMID: 458550

[ref106] Wilms L. Oberfeld D. (2018). Color and emotion: effects of hue, saturation, and brightness. Psychol. Res. 82, 896–914. doi: 10.1007/s00426-017-0880-8, PMID: 28612080

[ref107] Wilson G. Brewster S. A. (2017). “Multi-moji: combining thermal, Vibrotactile & Visual Stimuli to expand the affective range of feedback”. in *Proceedings of the 2017 Conference on Human Factors in Computing Systems (CHI ‘17)*. pp. 1743–1755.

[ref9010] Wilson D. Sperber D. (2006). Relevance theory. In The handbook of pragmatics. (Eds.) Horn L. Ward G. (Oxford: Blackwell), 606–632. doi: 10.1002/9780470756959.ch27

[ref9011] Yap D. F. So W. C. Melvin Yap J. M. Tan Y. Q. Teoh R. L. S. (2011). Iconic gestures prime words. Cognitive science, 35, 171–183. doi: 10.1111/j.1551-6709.2010.01141.x 21428996

[ref90031] Zhang Z. Alvina J. Héron R. Safin S. Détienne F. Lecolinet E. (2021). “Touch without Touching: Overcoming Social Distancing in Semi-Intimate Relationships with SansTouch“. in Proceedings of the 2021 CHI Conference on Human Factors in Computing Systems. 651, 1–13.

[ref108] Zhang Z. Héron R. Lecolinet E. Detienne F. Safin S. (2019). “VisualTouch: enhancing affective touch communication with multi-modality stimulation”. in *Proceedings of the 2019 International Conference on Multimodal Interaction (ICMI ‘19)*. pp. 114–123.

