# Supplementary material for: Touching at a distance: the elaboration of communicative functions from the perspective of the interactants
Source: Front Psychol. 2024 Dec 18;15:1497289. doi: 10.3389/fpsyg.2024.1497289 (PMC11689214; doi:10.3389/fpsyg.2024.1497289)
Supplement: Supplementary file 1 [file Table_1.DOCX]

Supplementary Material

Video 1 – Visual Touch use demonstration

Video 2 – Excerpt of the interaction of a couple

Supplementary Table 1 - Codebook of the functions of visual-touch used in interaction

| FUNCTIONS | | **DESCRIPTORS** | **EXAMPLES** |
| --- | --- | --- | --- |
| Interaction management | Turn-taking | The participants evoke using the device to take the turn. | - not reported in interview- |
|  | Backchannel | The participants talk about how visual-touch is used when listening to the speaker. | “It’s as if you are talking and the person who is not talking is sending you signals like ‘mmh’, ‘okay’ or nodding.” |
|  | Turn-giving | The participants evoke that the visual-touch is used to end the message, to explain that they have nothing further to add. | “I see that as with text message. It was the end of the text. Like the last word.” |
|  | Beat | The participants discuss about how the use of the device help them segment and add rhythm to their speech. | - not reported in interview- |
| Meaning Making | Emphasis | The participants evoke how they use the device when they felt or express a strong feeling. | “It was when I think we had felt something strong. The both of us, we would send some little spikes to notify.” |
|  | Referring | The participants refer to when they used the device to express that they agreed on what they were talking about, the subject or the facts. | “It’s when we agreed on what we were talking about. Well I think. Sometimes we were on the same line and it was to signify that thing.” |
|  | Modulating | The participants evoke visual-touch as a way to modulate their tone, being playful, ironic or apologetic for instance. | “I repeated it but it was like saying I like your blue eyes while teasing you. It was a little tease.” |
|  | Illustration | The participants discuss the iconic aspects of their use of the device. | “At the end to represent the timeline of the event, I kind of drew a timeline, a horizontal line with vertical bars.” |
| Relationship maintenance | Positive Affect | The participants explain how they use the device to convey positive affect such as love, tenderness, support. | “It is her negative memory and she uses the right colour, but I keep using a clear colour. […] It was to, as I know how she’s feeling, to caress her […] comforting her.” |
|  | Negative Affect | The participants explain how they use the device to express negative affect such as annoyance, sadness, oppression. | “There is the ‘a lot’, the ‘too much’ when I touch the whole screen to express the feeling of heaviness” |
|  | Closeness | The participants talk about the use of the device to maintain a sense of closeness between them. Touching is often explained as mimed caressing. | “I think that the device replaced the person. To know that he would receive the sensation. […] As when I will hug him.” |
|  | Play  (Mimicry) | The interactants play with the device. This can be a question-and-answer game with the device, or to repeat the partner’s touch. | “I think it’s something like that, a response. She’s using it [the device] and I will want to use it.” |
| Adaptors (Doodle) | | The participants evoke using the device as they would manipulate a pen or a rubber band or scratch themselves for example. | “No it wasn’t in response. It’s as if I was playing with a rubber band, I turn my hands.” |

Supplementary Table 2 - Codebook of the indicators used by the participants to elaborate the functions

| **INDICATORS** | | **DESCRIPTORS** | **EXAMPLES** |
| --- | --- | --- | --- |
| TACTILE | pattern | The participants talk about general forms or directions of their visual-touch | “When we were lost it was more like lines that we did with our fingers.” |
|  | cadence | The participants talk about the rhythmic characteristics of their touch | “The touch is frenetic […] I was telling the story and there was something a little frenetic and exciting in the story and the more I used the device.” |
| VISUAL | colour | The participants discuss the colours and their meaning | “It is her negative memory and she uses the right colour, but I keep using a clear colour. […] It was to, as I know how she’s feeling, to caress her […] comforting her.” |
|  | illustrative aspects | The participants talk about iconic drawing on the smartphone | “At the end to represent the timeline of the event, I kind of drew a timeline, a horizontal line with vertical bars.” |
| VERBAL | explication | The participants note that they gave voiced explanation of the meaning or of one physical aspect (visual or tactile) of the produced stimuli | “Yes, we talked about it at the beginning during the familiarisation phase. And I told him ‘*did you noticed when I did that to comfort you? Something soft as a caress on the arm?’* So to reuse it I thought he would understand.” |
|  | theme | The participants express how the verbal content and their knowledge of the memory played a role the understanding of the function | “It was when I think we had felt something strong. The both of us, we would send some little spikes to notify.” |
| PARAVERBAL | alignment & prosody | The participants discussed how the alignment between multiple modalities helped in understanding the meaning | “Yes I'd been paying attention, you see it was a little red dot and the white thing and then it really follows the rhythm of your voice.” |
|  | interaction role | The participants discussed the way their role (listener or speaker) influenced their use or understanding of visual-touches | “I really think there is a link with the first one. I am comforting myself when I talk. When he’s speaking it’s more like I’m going to hug him” |
|  | interaction time | The participants discussed how the timing of the interaction, notably knowing that it was the end of a subject, plays a role in the understanding of visual-touches | “I was trying to convey the reactions you'd normally have in a conversation, but through touch” |
| NONVERBAL | gaze | The participant talked about the orientation of the eyes to understand the touch, notably its orientation | “when I want to communicate something are the times when I'm looking at him.” |
|  | facial expression | The participants evoke the facial expression as influencing the perception of touch | “she's looking at me a bit like that [with tenderness] and she's also using the device at the same time” |
|  | mimicry | The participants notice their synchronicity regarding tactile or visual aspects | “We noticed that we do exactly the same patterns again, and at the end it was very close in terms of colours. But we couldn’t say why.” |
